# Exploring the Interaction between the SWI/SNF Chromatin Remodeling Complex and the Zinc Finger Factor CTCF

**DOI:** 10.3390/ijms21238950

**Published:** 2020-11-25

**Authors:** Mariangela Valletta, Rosita Russo, Ilaria Baglivo, Veronica Russo, Sara Ragucci, Annamaria Sandomenico, Emanuela Iaccarino, Menotti Ruvo, Italia De Feis, Claudia Angelini, Sara Iachettini, Annamaria Biroccio, Paolo Vincenzo Pedone, Angela Chambery

**Affiliations:** 1 Department of Environmental, Biological and Pharmaceutical Sciences and Technologies, University of Campania Luigi Vanvitelli, 81100 Caserta, Italy; mariangela.valletta@unicampania.it (M.V.); rosita.russo@unicampania.it (R.R.); ilaria.baglivo@unicampania.it (I.B.); veronica.russo@unicampania.it (V.R.); sara.ragucci@unicampania.it (S.R.); 2 Istituto di Biostrutture e Bioimmagini IBB, National Research Council, 80134 Napoli, Italy; annamaria.sandomenico@gmail.com (A.S.); emanuela.iaccarino@gmail.com (E.I.); menotti.ruvo@unina.it (M.R.); 3 Istituto per le Applicazioni del Calcolo IAC ‘M. Picone’, National Research Council, 80131 Napoli, Italy; i.defeis@iac.cnr.it (I.D.F.); c.angelini@iac.cnr.it (C.A.); 4 Oncogenomic and Epigenetic Unit, IRCCS-Regina Elena National Cancer Institute, 00144 Roma, Italy; sara.iachettini@ifo.gov.it (S.I.); annamaria.biroccio@ifo.gov.it (A.B.)

**Keywords:** CTCF, SWI/SNF, BRG1, mass spectrometry, protein–protein interaction, BRK, transcription factor, chromatin

## Abstract

The transcription factor CCCTC-binding factor (CTCF) modulates pleiotropic functions mostly related to gene expression regulation. The role of CTCF in large scale genome organization is also well established. A unifying model to explain relationships among many CTCF-mediated activities involves direct or indirect interactions with numerous protein cofactors recruited to specific binding sites. The co-association of CTCF with other architectural proteins such as cohesin, chromodomain helicases, and BRG1, further supports the interplay between master regulators of mammalian genome folding. Here, we report a comprehensive LC-MS/MS mapping of the components of the switch/sucrose nonfermentable (SWI/SNF) chromatin remodeling complex co-associated with CTCF including subunits belonging to the core, signature, and ATPase modules. We further show that the localization patterns of representative SWI/SNF members significantly overlap with CTCF sites on transcriptionally active chromatin regions. Moreover, we provide evidence of a direct binding of the BRK-BRG1 domain to the zinc finger motifs 4–8 of CTCF, thus, suggesting that these domains mediate the interaction of CTCF with the SWI/SNF complex. These findings provide an updated view of the cooperative nature between CTCF and the SWI/SNF ATP-dependent chromatin remodeling complexes, an important step for understanding how these architectural proteins collaborate to shape the genome.

## 1. Introduction

The ubiquitously expressed CCCTC-binding factor (CTCF) is a highly conserved transcription factor carrying 11 zinc finger domains involved in multiple DNA sequence-specific recognition [1,2,3]. Combinatorial use of zinc fingers is also responsible for CTCF pleiotropic functions including transcriptional activation/repression, insulator activity, and regulation of genomic imprinting. The number of CTCF-interacting proteins is constantly growing given their impact on understanding CTCF multiple functions and its role as a genome master regulator [1,4,5]. A suggestive hypothesis on CTCF-interacting proteins proposes that specific subsets of partners are recruited for assisting CTCF to perform distinct functions and that each of these complexes operates in a specific biological context [1,4]. Such a hypothesis would also effectively explain the high flexibility of CTCF in binding thousands of genomic sites acting as a transcriptional activator, repressor, and insulator [2,3,6]. Indeed, the CTCF protein partner network appears to be essential to the fine-tuning of sometimes opposite regulatory functions carried out by this highly conserved zinc finger protein. Several studies have also supported the idea that CTCF attracts different partners in both a tissue and genomic context-specific manner, further amplifying the diversity of CTCF roles [5].

Several chromatin proteins, including both structural proteins and enzymes, DNA-binding proteins, as well as other multifunctional proteins, interact with CTCF [4]. Among transcription factors, YB1 has been reported to interact with CTCF and to cooperate in Myc transcriptional repression, while Yy1 is involved with CTCF in Tsix transactivation [7,8]. CTCF has also been reported to interact with Kaiso, a member of the poxvirus and zinc finger transcription factors, at the human 5′ beta-globin insulator [9,10]. Moreover, CTCF also directly interacts with RFX and CIITA, a transcriptional coactivator that regulates gene expression by recruiting other transcription factors and chromatin remodelers [11,12]. Chromatin proteins including structural proteins (e.g., H2A and H2A.Z, CP190, and cohesin), enzymes (e.g., CHD7, CHD8, PARP1, and topoisomerase II) and histone-modifying proteins (e.g., Sin3 and Taf1/Set) have been identified as CTCF cofactors and are reviewed by Zlatanova and Caiafa [4]. Moreover, CTCF has also been shown to interact with the nuclear-matrix phosphoprotein nucleophosmin [6] and with the large subunit of RNA pol II [13], relevant associations for its insulator and transcriptional regulation functions, respectively. Interestingly, a direct protein–protein interaction has been demonstrated to occur between the zinc finger domains of CTCF and the regions containing the two BRK domains of CHD7 and CHD8 helicases [14,15].

We recently contributed to expand the current knowledge of the human CTCF interactome by applying a high-throughput approach based on affinity purification and high-resolution LC-MS/MS analysis [16]. Large-scale identification of CTCF-binding partners allowed us to define BRG1, the major ATPase subunit of the chromatin remodeling switch/sucrose nonfermentable (SWI/SNF) complex, as a new partner of the transcriptional regulator CTCF, thus, establishing a relationship between two master regulators of chromatin architecture [16].

Within cells, BRG1 constitutes the catalytic subunit of distinct multiprotein complexes involved in DNA remodeling, transcriptional regulation, DNA replication, repair, and recombination [17]. The alteration of chromatin structure by chromatin remodeling complexes is considered to be a crucial step in the transcriptional regulation of eukaryotic genes [18]. In addition to the well characterized SWI/SNF chromatin remodeling complexes (i.e., BAF, PBAF and ncBAF), BRG1 can associate with numerous chromatin-modifying complexes such as WINAC, NUMAC, NCoR, and mSin3A/HDAC with different roles in transcriptional activation and repression [17]. Although these complexes share some subunits with SWI/SNF- and ISWI (Imitation SWItch) -based chromatin remodeling complexes, they are characterized by distinct organizational module composition [17].

Here, we investigated, by high-resolution nano-liquid chromatography coupled to electrospray ionization—tandem mass spectrometry (nano-LC-ESI-MS/MS), the presence of other components of the SWI/SNF chromatin remodeling complex in the human CTCF interaction network, identifying with high confidence 14 SWI/SNF protein subunits. Selected subunits of the SWI/SNF complex were further validated by Western blot and ChIP-seq analyses. We also investigated the direct interaction between CTCF and BRG1 and we found that the BRK-BRG1 domain binds the zinc finger motifs 4–8 of CTCF, thus, suggesting that these domains mediate the interaction of CTCF with the SWI/SNF complex.

This study expands the CTCF interactome scenario with many SWI/SNF factors, paving the way to future studies aimed at further elucidating the interplay between SWI/SNF remodeling complex and CTCF in the context of genome architecture.

## 2. Results

### 2.1. Immunoprecipitation and Identification of CCCTC-Binding Factor (CTCF)-Interacting Members of the Switch/Sucrose Nonfermentable (SWI/SNF) Complex

We previously generated a WiT49 cell line stably overexpressing the full-length CTCF to enable the purification and identification of CTCF-interacting complexes by affinity purification and untargeted high-resolution LC-MS/MS analysis [16]. By this approach, coupled to independent immunoprecipitations of endogenous proteins, we defined BRG1 as a new partner of the transcriptional regulator CTCF [16]. Now, in contrast to the previous broad screening of CTCF interactors, we have investigated the presence of other components of the SWI/SNF chromatin remodeling complex in the CTCF interactome. To this aim, protein identifications was inferred from a set of peptide-to-spectra matches (PSM) by assigning fragment ion mass spectra to peptide sequences by using database search engines to query at first a customized protein FASTA database containing all subunits of the human SWI/SNF complex and their isoforms.

**Figure 1:**
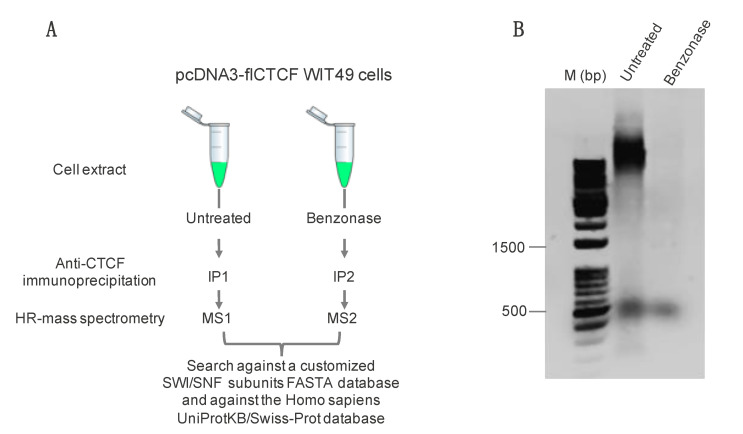
(**A**) Schematic representation of the strategy employed for the immunoprecipitation and identification of CCCTC-binding factor (CTCF)-interacting members of the switch/sucrose nonfermentable (SWI/SNF) complex. IP, immunoprecipitation; MS, mass spectrometry; (**B**) Agarose gel with DNA from untreated or Benzonase-treated cell extracts, as indicated. M, DNA size markers.

Since we were interested in proteins establishing protein-mediated interactions with CTCF and not those possibly co-purified via chromatin, AP-MS experiments were performed on extracts pretreated with the DNA/RNA digesting enzyme Benzonase, using as control untreated samples (Figure 1A). Addition of Benzonase completely removed chromatin/DNA from the untreated as compared with the Benzonase-treated extract (Figure 1B).

Protein complexes were purified by immunoprecipitation and subsequent protein-A affinity pulldown by using a method optimized as compared with that described by Marino et al. [16]. Samples were analyzed by mass spectrometry following tryptic digestion to identify the specific SWI/SNF complex subunits present in the samples. By applying stringent validation criteria, also based on a target-decoy PSM validation-based strategy (see Methods), 14 members of the SWI/SNF remodeling complex were identified with high confidence (Table 1). Subsequently, in order to assess that no bias or errors in the false discovery rate (FDR) calculation were introduced using a small database, identifications of SWI/SNF subunits were also substantially confirmed by searches against the Homo sapiens UniProtKB/Swiss-Prot database. Details of the identifications performed against both databases are reported in Tables S1 and S2. Most SWI/SNF members were commonly identified in the presence and absence of Benzonase treatment (Table 1) suggesting, as previously demonstrated for BRG1 [16], that the interaction between the proteins is independent from DNA and RNA.

**Figure 2:**
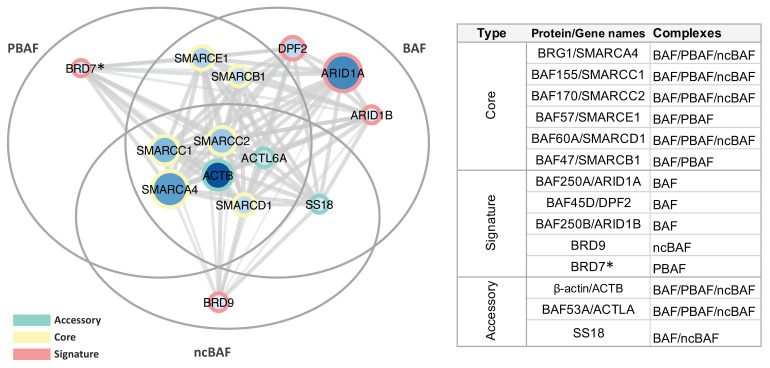
Cytoscape interaction network of members of the SWI/SNF remodeling complex identified by nano-LC-MS/MS in the CTCF IP. Node size and color intensity are related to the number of peptides identified by MS, while edge thickness is related to StringApp interaction confidence scores. Network clustering was performed according to the modular assembly of identified subunits in the alternative mammalian SWI/SNF (mSWI/SNF) complexes. On the right, is reported the classification and alternative protein/gene names of identified SWI/SNF core, signature, and additional accessory subunits and their presence within the following three classes of mammalian SWI/SNF complexes: canonical BRG1/BRM-associated factor (BAF), polybromo-associated BAF (PBAF), and ncBAF complexes [19]. The asterisk indicates a single peptide-based identification in both Benzonase-treated and untreated samples.

A clusterization of identified proteins according to the architecture of the three classes of mammalian SWI/SNF complexes, i.e., canonical BRG1/BRM-associated factor (BAF), polybromo-associated BAF (PBAF), and newly defined ncBAF complex [19]—is reported in Figure S1, revealing the presence in the CTCF immunoprecipitation (IP) of core, accessory, as well as signature subunits belonging to the three complexes. These subunits were also mapped on a StringApp cytoscape interaction network showing the modular assembly of identified subunits within the alternative BAF, PBAF, and ncBAF mammalian SWI/SNF (mSWI/SNF) complexes (Figure 2).

In particular, we confirmed our previous findings reporting BRG1 as one of the main SWI/SNF component present in the CTCF interactome, since it was identified with a high number of PSM (Figure S2A–C) at a ppm accuracy level between theoretical and experimental molecular masses (Figure S2B). In addition, we identified three subunits of the so-called core module required as platform for BAF, PBAF, and ncBAF formation (i.e., BAF155, BAF170, and BAF60A, reported in yellow in Figure 2). We also identified two additional core subunits not reported to be part of the ncBAF complex (i.e., BAF57 and BAF47). Base peak chromatograms showing the retention times of peptides mapped on selected core SWI/SNF subunits are reported in Figure S3A (BAF155) and Figure S3B (BAF170). Representative MS/MS spectra of peptide mapping on BAF155 and BAF170 are reported in Figures S4 and S5, respectively, while representative MS/MS spectra of peptide mapping on all the other SWI/SNF subunits are reported in Figures S6–S12. 

**Figure 3:**
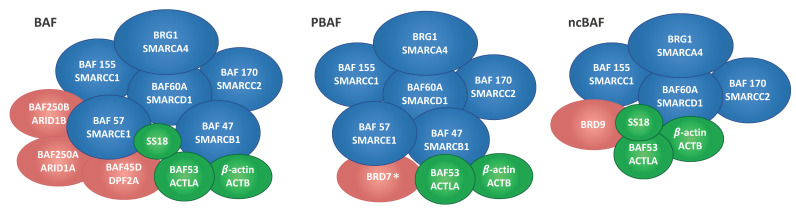
Schematic representation of the BAF, PFAF, and ncBAF SWI/SNF complexes summarizing the core subunits (blue) and additional accessory subunits (green) identified by nano-LC-MS/MS in the CTCF IP. Signature subunits defining each individual complex are reported in red. The asterisk indicates a single peptide-based identification.

Ten accessory and signature subunits of specific SWI/SNF complexes, reported respectively in green and red in Figure 2, were also present in the CTCF IP. Of these, the BAF-specific AT-rich interactive domain containing protein 1 (ARID1A) was the member identified with the highest number of peptides. Notably, all subunits reported to be part of the BAF complex were identified by our MS approach [19], while we found one signature subunit specific to the ncBAF (i.e., BRD9) complex [19]. In addition, only a single peptide was mapped on BRD7, a signature PBAF-specific subunit [19]. Although MS/MS spectra for this peptide were identified in both Benzonase-treated and untreated samples, we could not exclude the possibility of a false positive identification and the interaction of BRD7 with CTCF needs further confirmation. Published molecular associations reported in some studies for PBAF (i.e., ARID2, PH10, and PB1) and ncBAF (i.e., GLTSCR1) complexes may have not been detected because based on transient or of low affinity interactions, mediated by indirect protein–protein interactions, or dependent on specific biochemical conditions. Overall, our MS data provide evidence of the presence of the complete architectural framework for the mSWI/SNF chromatin remodeler complex family in the CTCF pulldown (Figure 3). In addition to BRG1 [16], none of these proteins were previously identified as binding partners of CTCF.

### 2.2. Validation by Western Blot

**Figure 4:**
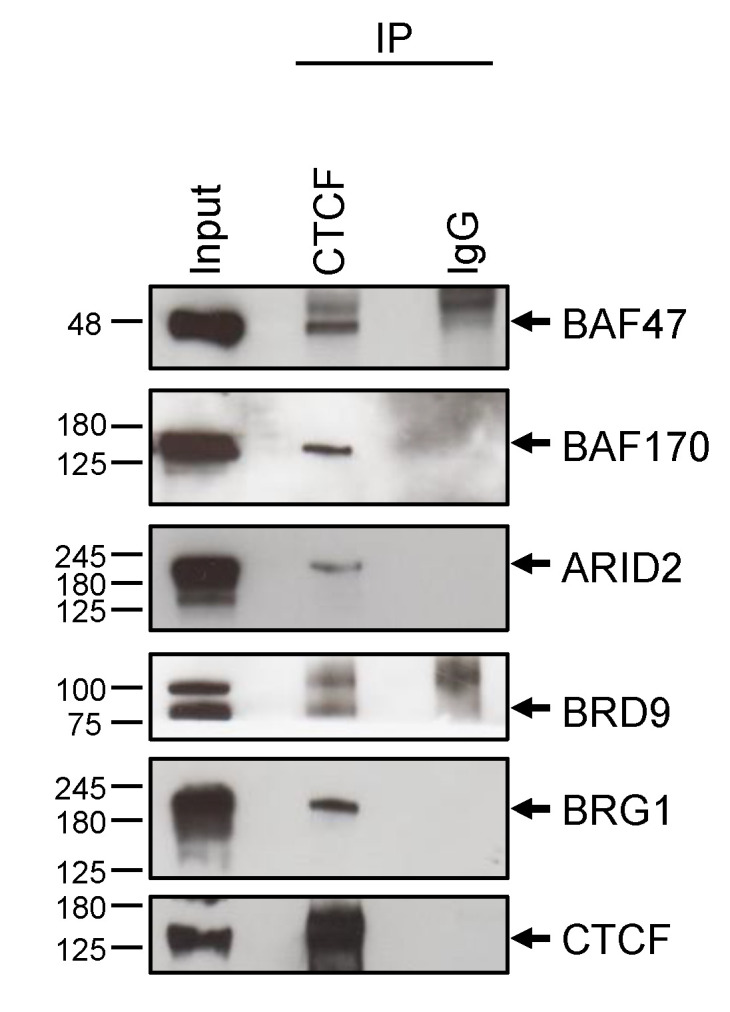
Validation of CTCF interactors by Western blot. Endogenous CTCF-immunoprecipitated samples from HeLa nuclear cell lysates were blotted and probed with anti-BRG1, anti-BRD9, anti-ARID2, anti-BAF170, and anti-BAF47 antibodies. Anti-CTCF was used as a positive control. Control IP by rabbit IgG and 5% input are also shown.

To validate our MS-based affinity pull-down approach and further confirm the interaction of CTCF with SWI/SNF complexes with a complementary experimental approach, we purified endogenous CTCF from whole cell lysate of HeLa cells by immunoprecipitation followed by immunoblot using antibodies against subunits of the SWI/SNF complex (Figure 4).

In addition to confirming the presence of BRG1 in the CTCF IP, we selected representative core and signature subunits. In particular, we find back the two core subunits, BAF170 and BAF47. Given the high confidence of identification of signature subunits of the BAF complex, mapped with a very high number of peptides (33 and 10 peptides for the ARID1A and the DPF2 subunits, respectively), we decided to validate the presence in HeLa cells of signature PBAF and ncBAF subunits; in particular, we selected the ARID2 subunit, not identified by our MS approach, and the BRD9 subunit, respectively. We confirmed in the CTCF IP from HeLa cells, the presence of the signature subunits ARID2 and BRD9. These results confirm specific interactions of the selected SWI/SNF candidate proteins with CTCF, further supporting AP-MS data.

### 2.3. Genomic Co-Occupancy by CTCF and SWI/SNF Subunits

**Figure 5:**
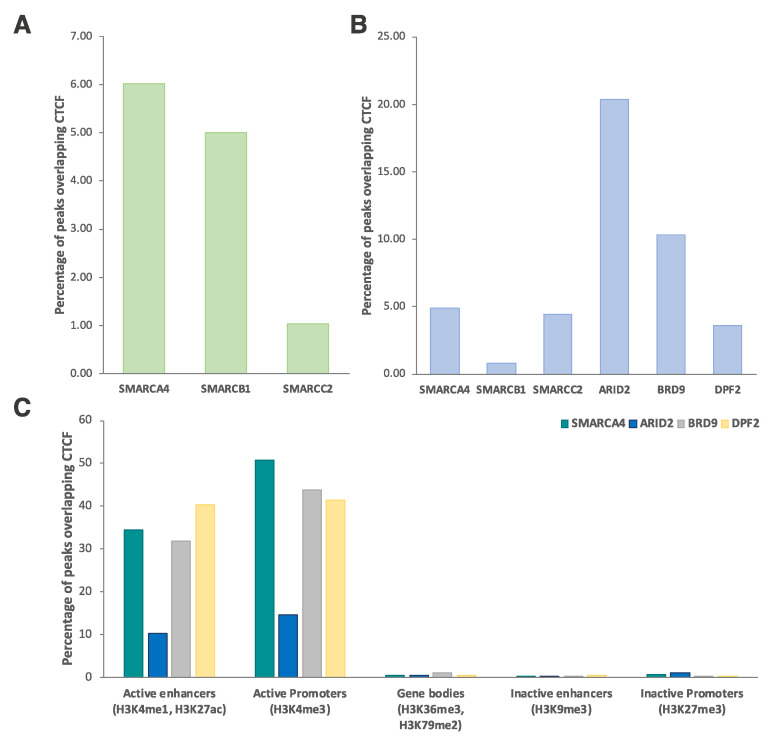
Percentages of peaks from ChIP-seq experiments for each SWI/SNF subunit overlapping CTCF ChIP-seq peaks in HeLa-S3 (**A**) and K562 (**B**) cell lines; (**C**) Bar charts reflecting percentages of the number of SWI/SNF subunit peaks overlapping CTCF with the histone markers associated with the specified chromatin features in K562 cells.

To probe genome-wide overlap of CTCF with SWI/SNF complexes, we first analyzed the HeLa-S3 cell line for which the only published ChIP-seq datasets simultaneously available along with CTCF were those of the two SWI/SNF core subunits SMARCB1 and SMARCC2 and of the ATPase SMARCA4. In this dataset, we found overlapping peaks between CTCF and SMARCA4 (6%), SMARCB1 (5%) and SMARCC2 (1%) (Figure 5A).

Next, we focused on K562 cell line by confirming that about 5% of both SMARCA4 and SMARCC2 sites overlap with CTCF (Figure 5B). A lower overlap was also found for SMARCB1, likely depending on the small number of consensus peaks initially detected (Figure 5B).

Furthermore, in K562 cells, ChIP-seq datasets were also concurrently available for CTCF and the members ARID2, BRD9, and DPF2 as representative signature subunits of the PBAF, ncBAF, and BAF complexes, respectively. With variable percentages of overlapping sites, we found colocalizations of these subunits with CTCF (Figure 5B), with the highest number of overlapping sites shared by ARID2 and CTCF (20%), followed by BRD9 (10%) and DPF2 (3%).

Overall, even considering the high variability and data quality issues affecting the ability to compare data from multiple studies, pairwise comparisons revealed a genomic co-occupancy by CTCF and SWI/SNF subunits, further supporting our MS results and suggesting functional implications for these interactions.

Subsequently, in the K562 cell line, we sought to investigate if a different SWI/SNF assembly, together with CTCF, was associated with specific chromatin features. Therefore, we analyzed the overlap of CTCF-ARID2, CTCF-BRD9, and CTCF-DPF2 colocalized regions, as representative signature subunits of the PBAF, ncBAF, and BAF complexes, respectively, with specific chromatin markers. In particular, colocalizations were evaluated with markers associated with active enhancers (H3K4me1, H3K27ac), active promoters (H3K4me3), gene bodies (H3K36me3, H3K79me2), inactive enhancers (H3K9me3), and inactive promoters (H3K27me3). SMARCA4 was also included in this analysis as a representative member present in all the three complexes. We found that all SWI/SNF subunits highly overlap with CTCF at active enhancers and promoters (Figure 5C). In agreement with previous findings on SMARCA4 and CTCF co-occupancy [16], we conclude that the representative members of PBAF, ncBAF, and BAF SWI/SNF complexes overlap with CTCF on transcriptionally active chromatin regions.

### 2.4. CTCF Directly Interacts with the BRK Domain of BRG1

**Figure 6:**
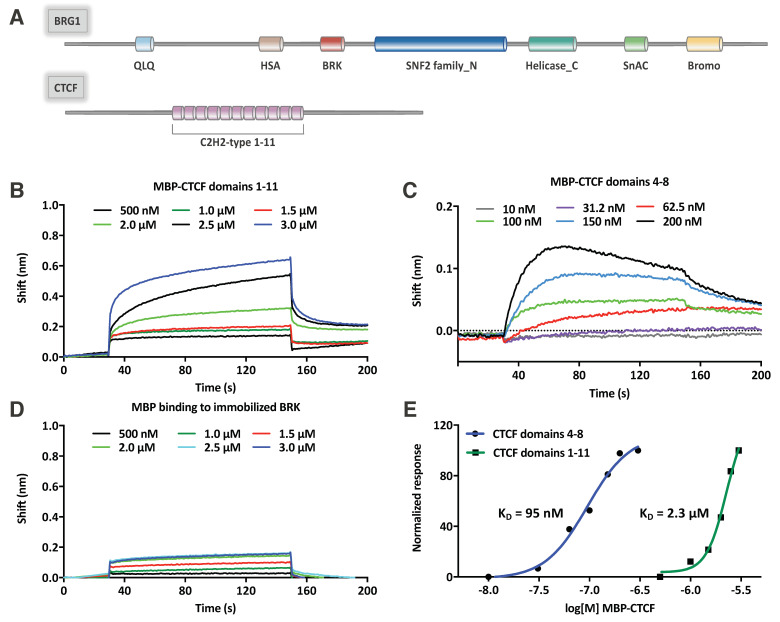
Binding studies between the BRK domain of BRG1 and recombinant CTFC zinc finger domains by biolayer interferometry (BLI). Experiments were performed using the synthetic BRK domain immobilized on the sensor tip and using the CTFC zinc finger domains as soluble analytes. (**A**) Schematic representation of BRG1 and CTCF domains organization; (**B**) Overlaid interferograms obtained for the binding of maltose-binding protein (MBP)-fused CTCF domains 1–11; (**C**) Overlaid interferograms obtained for the binding of MBP-fused CTCF domains 4–8; (**D**) Overlaid interferogram obtained using MBP alone; (**E**) Normalized binding curves obtained plotting the maximum BLI shifts achieved in the various experiments, minus the MBP contribution, versus the analyte concentrations.

Although our findings demonstrate that CTCF coassociates with SWI/SNF members, further investigations are required to determine whether this interaction is direct or mediated by other proteins. We hypothesized that a direct interaction between CTCF and BRG1 was likely to occur given the high number of peptides identified in the CTCF IP and that it could be mediated by the recognition between protein domains commonly present in known CTCF interactors. Thus, we focused our attention on the BRK domain located between the HSA domain and the ATPase module of BRG1 (Figure 6A) for which a potential role in protein–protein interaction has been recently reported [20]. In addition, this sequence motif is conserved in other chromatin remodeling enzymes such as the chromodomain helicases CHD7 and CHD8. For both these proteins, it has been shown that regions containing their two BRK domains are able to interact with CTCF zinc finger domains (Figure 6A) [14,15]. In order to test whether the BRK domain of BRG1 could also directly bind to CTCF, we prepared, by chemical synthesis, the 45 amino acids long polypeptide corresponding to the region 612–656 of BRG1 (BRK domain of BRG1, Figure 6A). The polypeptide was obtained with a final yield of about 5%, as estimated considering the starting synthesis scale, and was purified to homogeneity by RP-HPLC (purity > 97%, Figure S13A). Peptide identity was confirmed by mass spectrometry analysis (Figure S13B). Then, we produced recombinant maltose-binding protein (MBP)-fused CTCF zinc finger domains 1–11 (residues 263–582) and 4–8 (residues 348–494) in a bacterial expression system for probing the binding of BRK-BRG1 to different regions of CTFC by biolayer interferometry (BLI). Experiments were performed immobilizing the synthetic domain on a BLI sensor chip and probing the binding by exposing it to solutions of the zinc finger domain solutions at different concentrations. The BLI experiments showed that both zinc finger regions of CTCF interact with the BRK domain, suggesting that the CTCF zinc fingers 4–8 are mostly involved in the interaction (Figure 6). Indeed, while the fragment 263–582, containing the zinc fingers 1–11 bound the polypeptide in the micromolar range (Figure 6B, 0.5 µM ÷ 3.0 µM), the shorter variant containing the zinc fingers 4–8 was able to interact in the nanomolar range (Figure 6C, 10 nM ÷ 200 nM). The presence of MBP did not substantially affect the binding (Figure 6D). By plotting the maximum BLI shift values achieved in the various experiments, minus the MBP contribution, versus the protein’s concentrations (Figure 6E), we could obtain an estimation of the different dissociation constants (KDs) underlying the interactions. The estimated KDs were around 2.3 µM for domains 1–11 and around 95 nM for the domains 4–8 (Figure 6E).

## 3. Discussion

An important architectural role of CTCF in three-dimensional (3D) genome conformation and assembly has recently emerged [21,22,23,24]. In this context, CTCF is also recognized as an essential component of the highly self-interacting regions known as topological associated domains (TADs) in cooperation with other regulators of genome topology such as the well-studied ring-shaped protein complex cohesin [21,25,26,27,28]. The role in genome architecture also suggests a crosstalk between CTCF and other factors involved in chromatin remodeling.

We recently reported evidences of a physical co-association of CTCF with BRG1 [16]. Here, we set out to build on our previous work by expanding the CTCF interactome with many SWI/SNF protein subunits and our experimental setup suggests that these interactions are independent of chromatin. Our findings provide the missing biochemical evidence of co-association of CTCF with the SWI/SNF complex, which to date has only been investigated by genome-wide mapping approaches [29,30,31]. Indeed, cooperation between CTCF and the SWI/SNF ATP-dependent remodeling complexes as master regulators of genome topology has long been postulated. Because maintenance of TAD boundaries is affected by SMARCA4, and by extension the SWI/SNF complex, previous reports have showed that SMARCA4 and other subunits of the mammalian SWI/SNF bind near regions critical for genome organization such as CTCF binding sites [29,30,31]. According to these observations, Baructu and co-workers demonstrated that BRG1 knockdown affected long-range genomic interactions resulting in a reduction of TAD boundary strength, likely depending on nucleosome occupancy by BRG1 around CTCF sites [25,31]. In particular, intersection of CTCF ChIP-seq dataset MCF-10A [32] with SMARCA4 peaks resulted in an overlap of 10% of all SMARCA4 peaks with CTCF, implying a crosstalk between SMARCA4 and CTCF for a subset of bound genomic regions [31].

In addition to the cooperation in spatial organization of chromatin topology, CTCF and SWI/SNF association may be particularly relevant in transcriptional regulation, also considering that SWI/SNF and BRG1 are activators and repressors of many genes [17,33]. These multiple functions are mediated by interactions of BRG1 with diverse nuclear proteins including nuclear receptors, chromatin proteins, and proteins involved in genomic maintenance [17]. Moreover, targeting of SWI/SNF complexes to gene-specific promoters has been suggested to be assisted by transcriptional factors or histone-binding domains [34,35,36]. In particular, BRG1-containing complexes are selectively recruited on distinct sets of promoters through association with zinc finger proteins. This cooperation increases DNA accessibility by generating an open chromatin conformation at gene promoters and enhancers, thus, favoring the transcription factor binding to site-specific sequences [36,37]. In agreement with the scenario depicting SWI/SNF and transcription factor cooperation important for transcriptional activation, we also found that members of the SWI/SNF complex identified by MS, overlapped with CTCF on transcriptionally active chromatin regions. Similarly, Michel et al., by a comprehensive mapping of SWI/SNF assemblies on chromatin, found that the ncBAF complexes uniquely localized to CTCF sites and promoter-proximal sites [30]. Taken together, these data support a view in which CTCF and SWI/SNF complexes converge into active chromatin hubs creating conformations favorable for transcription.

Among previously reported CTCF-binding partners, other chromatin remodelers regulating transcription have been identified. In particular, previous investigations have shown that CTCF directly interacted through zinc finger domains with members of the chromodomain helicase DNA-binding family (i.e., CHD7 and CHD8). For CHD8, a direct role in CTCF-mediated intra-chromosomal contacts has been demonstrated [14]. A distinctive structural feature of CHD proteins is the presence of BRK domains at the C-terminus of the ATPase domain.

As outlined previously, it has been shown that the region of CHD8 containing its two BRK domains interacts with the zinc finger domains of CTCF [14]. Similar binding capabilities were also shown for the GST-fused CTCF zinc finger domain (residues 260–586) and the BRK domains of CHD7 [15]. The BRK domain is a 50 amino acids module of unknown function that interestingly is also conserved in the brahma/BRG1 family of chromatin remodeling enzymes [38]. Here, we show that the BRK-BRG1 domain binds the CTCF region including the eleven zinc finger domains and that the region encompassing domains from 4 to 8 strongly contributes to this interaction. Interestingly, the same five domains were previously shown to be essential for strong DNA binding and for the recognition of the core DNA sequence common to most CTCF sites [39,40,41]. The model of alternative involvement of DNA-binding zinc fingers in protein–protein interactions is gaining increasing interest [7,42]. In support of this model, our results provide evidence that the zinc finger domains can mediate both DNA binding and functionally important protein–protein interactions, as also demonstrated for other CTCF-interacting proteins such as Sin3A and YB-1 [7,43].

Because both CTCF and chromatin remodelers are engaged in multiple interactions with other proteins, one obvious but critical question for future studies is whether the association between CTCF and BRG1 is mutually exclusive with respect to other CTCF interacting proteins such as cohesin. Cohesin promotes the folding of the genome into loops that are progressively enlarged [44,45,46,47] and that are anchored by CTCF [48]. Recently, Li et al. provided crucial insights into the molecular mechanism underpinning the dynamic regulation of chromatin folding by cohesin and CTCF, demonstrating that a fragment within the CTCF N-terminus, not including the zinc finger domains, interacted with the SA2-SCC1 subunits of human cohesin [49]. This observation does not exclude that other proteins may contribute to modulate the ability of CTCF and cohesin to catalyze genome folding. According to the hypothesis of Ghirlando and Felsenfeld, the “loop extrusion” process would, for example, require an energy source, suggested to be an helicase, to propel cohesin along the loop [1]. Our observations are in agreement with a model where SWI/SNF, CTCF, and other proteins such as cohesin work in concert as architectural factors to regulate genome folding and gene expression.

## 4. Materials and Methods

### 4.1. Sample Preparation for Mass Spectrometry Analysis

WiT49 cell line stably overexpressing CTCF was obtained, as described in Marino et al. [16] and was cultured in ten 150 mm plates in Iscove’s Modified Dulbecco’s Media, supplemented with 10% fetal calf serum, 100 units/mL penicillin, and 100 mg/mL streptomycin at 37 °C in a humidified 5% CO_2_ atmosphere. Cells were harvested by trypsinization and washed with phosphate-buffered saline (PBS). For Benzonase digestion, cells were lysed in 10 mM Tris-HCl pH 7.4, 350 mM NaCl, 1 mM MgCl_2_, 1% Triton X-100, 10% glycerol and incubated for 30 min at room temperature in the presence or absence of 250 units of Benzonase (Sigma-Aldrich, St. Louis, MO, USA). Protein concentration was determined by Bradford assay. For immunoprecipitation (IP), protein lysates (2 mg) were diluted in IP buffer up to 1 mL (50 mM Tris-HCl pH 7.4, 150 mM NaCl, 0.25% Na-deoxycholate) and incubated for 1 h, at 4 °C, with DiaMag Protein A coated magnetic beads (40 µL, Diagenode, Seraing, Belgium). After the pre-clearing step, samples were incubated overnight at 4 °C with polyclonal anti-CTCF (Diagenode C15010210, 10 μg) and polyclonal rabbit anti-IgG (Diagenode C15410206, 10 µg) as negative control. Then, immunoprecipitated proteins were incubated for 3 h under rotation at 4 °C with the DiaMag protein A-coated magnetic beads (40 µL, Diagenode) pre-washed in the IP buffer. Beads were collected on a magnetic stand, washed three times with 100 µL of 100 mM NH_4_HCO_3_ pH 8.0, and resuspended in 100 µL of the same buffer. Proteins were reduced with 10 mM dithiothreitol (final concentration) at 55 °C for 1 h and, following a wash step with 100 µL of NH_4_HCO_3_, carbamidomethylated with 7.5 mM iodoacetamide (final concentration) at room temperature in the dark for 15 min. Following a further wash step with 100 µL of NH_4_HCO_3_, enzymatic hydrolyses were performed by the addition of 0.2 µg of tosyl phenylalanyl chloromethyl ketone (TPCK)-treated trypsin to the reduced and alkylated mixture. Digestions were performed by incubation at 37 °C for 3 h, followed by a further addition of 0.2 µg of fresh trypsin and incubation for 16 h. After digestions, samples were centrifuged at 10,000× *g* for 15 min and supernatants were dried under vacuum in a SpeedVac Vacuum (Savant Instruments, Holbrook, NY, USA). Then, samples were resuspended in 20 µL of H_2_O/TFA 2% and centrifuged at 10,000× *g* for 15 min. Aliquots of the supernatant (10 µL) were diluted 1:1 in H_2_O/TFA 2% and analyzed in triplicate (5 µL/injection) by high resolution nano-LC-tandem mass spectrometry [16].

### 4.2. High Resolution Nano-LC−Tandem Mass Spectrometry

Mass spectrometry analysis was performed on a Q-Exactive Orbitrap mass spectrometer equipped with an EASY-Spray nano-electrospray ion source (Thermo Fisher Scientific, Bremen, Germany) and coupled to a Dionex UltiMate 3000RSLC nano system (Thermo Fisher Scientific), as reported in [16]. Solvent composition was 0.1% formic acid in water (solvent A) and 0.1% formic acid in acetonitrile (solvent B). Peptides were loaded on a trapping PepMap™ 100 μCartridge Column C18 (300 μm × 0.5 cm, 5 μm, 100 Å) and desalted with solvent A for 3 min at a flow rate of 10 μL/min. After trapping, eluted peptides were separated on an EASY-Spray analytical column (15 cm × 75 μm ID PepMap RSLC C18, 3 μm, 100 Angstrom), heated to 35 °C, at a flow rate of 300 nL/min by using the following gradient: 4% B for 3 min, from 4% to 22% B in 50 min, from 22% to 35% B in 10 min, from 35% to 90% B in 5 min. A washing (90% B for 5 min) and a re-equilibration (4% B for 15 min) step was always included at the end of the gradient. Eluting peptides were analyzed on the Q-Exactive mass spectrometer operating in positive polarity mode with capillary temperature of 280 °C and a potential of 1.9 kV applied to the capillary probe. Full MS survey scan resolution was set to 70,000 with an automatic gain control (AGC) target value of 3 × 10^6^ for a scan range of 375−1500 *m*/*z* and maximum ion injection time (IT) of 100 ms. The mass (*m*/*z*) 445.12003 was used as lock mass. A data-dependent top 5 method was operated during which higher-energy collisional dissociation (HCD) spectra were obtained at 17,500 MS2 resolution with an AGC target of 1 × 10^5^ for a scan range of 200−2000 *m*/*z*, maximum IT of 55 ms, 2 *m*/*z* isolation width, and a normalized collisional energy (NCE) of 27. Precursor ions targeted for HCD were dynamically excluded for 15 s. Full scans and Orbitrap MS/MS scans were acquired in profile mode, whereas ion trap mass spectra were acquired in centroid mode. Charge state recognition was enabled by excluding unassigned and singly charge states.

### 4.3. MS Data Processing

The acquired raw files were analyzed with the Proteome Discoverer 2.1 software (Thermo Fisher Scientific) using the SEQUEST HT search engine. The HCD MS/MS spectra were searched against a custom database including all subunits of the human SWI/SNF complex and their isoforms as well as against the Homo sapiens UniProtKB/Swiss-Prot database (release 2019_11, 20380 entries), assuming trypsin (full) as digestion enzyme and two allowed number of missed cleavage sites. The mass tolerances were set to 10 ppm and 0.02 Da for precursor and fragment ions, respectively. Oxidation of methionine (+15.995 Da) and N-terminal acetylation (+42.011 Da) were set as dynamic modifications and carbamidomethylation of cysteine (+57.021 Da) as static modification. False discovery rates (FDRs) for peptide spectral matches (PSMs) were calculated and filtered using the Target Decoy PSM Validator node in Proteome Discoverer. The target-decoy PSM validator node specifies the PSM confidences on the basis of dynamic score-based thresholds [50]. It calculates the node-dependent score thresholds needed to determine the FDRs, which are given as input parameters of the node. The target-decoy PSM validator was run with the following settings: Maximum Delta Cn 0.05, a strict target FDR of 0.01, a relaxed target FDR of 0.05, and validation based on q-value. The protein FDR validator node in Proteome Discoverer was used to classify protein identifications based on q-value. Proteins with a q-value of < 0.01 were classified as high confidence identifications and proteins with a q-value of 0.01–0.05 were classified as medium confidence identifications. Only proteins identified with high confidence were retained with an FDR of 1%. The resulting list of SWI/SNF complex subunits identified in the CTCF affinity pulldown were filtered, according to criteria reported in [16] and the absence in control IgG IP sample (Table S3). Proteins identified by searching MS/MS spectra against a custom common contaminant database were also not considered. The mass spectrometry proteomics data have been deposited to the ProteomeXchange Consortium via the PRIDE partner repository with the dataset identifier PXD022037.

### 4.4. Bioinformatic Analyses

The bubble plot showing clusterization of proteins identified by LC-MS/MS according to the architecture of the three classes of mammalian SWI/SNF complexes (i.e., BAF, PBAF, and ncBAF) was performed through the ggplot2 package v 3.3.1 of the RStudio v 1.2.1335 environment for R (http://www.R-project.org), according to Mashtalir et al. [19] and based on classification of the SWI/SNF Infobase database (http://scbt.sastra.edu/swisnfdb/).

Interaction network of the SWI/SNF members identified by LC-MS/MS was constructed using the STRING database implemented in the StringApp plug-in for Cytoscape software 3.7.2 [51].

### 4.5. Validations by Western Blot Analyses

For the validation of selected subunits of the SWI/SNF complex, IP experiments were performed followed by Western blot analyses. Then, 2 × 10^6^ HeLa cervical cancer cells were plated in 150 mm plates and lysed, after 48 h, with buffer A (10 mM Hepes pH 7.9, 10 mM KCl, 0.1 mM EDTA, 0.1 mM EGTA, 0.6% NP-40, 1 mM DTT, and 1 mM PMSF) and buffer C (20 mM Hepes pH 7.9, 400 mM NaCl, 1 mM EDTA, 1 mM EGTA, 1 mM DTT, and 1 mM PMSF), completed with protease (ThermoScientific, Waltham, MA, USA) and phosphatase inhibitors (ThermoScientific), which resulted, respectively, in cytosolic and nuclear fraction isolation. Protein concentration was obtained by the colorimetric BCA protein assay (ThermoScientific) and 500 µg of nuclear cell fractions were processed for immunoprecipitation using 6 µg of the pAb anti-CTCF (Diagenode, C15410210-50) and 30 µL of Dynabeads Protein A and Protein G (Invitrogen, 10002D and 10004D, respectively), following manufacture’s instruction. The immunoprecipitation with the antibody against Normal Rabbit IgG (Santa Crutz, sc-3888) was used as negative control.

Subsequently, the immunoprecipitation products were fractionated by SDS-PAGE, transferred to a nitrocellulose filter, and subjected to immunoblot assay. The following primary antibodies were used for immunodetection: mAb anti-BAF47 (Ini1 (A-5)) (Santa Crutz, sc-166165); mAb anti-BAF170 (E-6) (Santa Crutz, sc-17838); mAb anti-ARID2 (E-3) (Santa Crutz, sc-166117); pAb anti-BRD9 (Bethyl, A303-781A-T); and pAb anti-BRG1 (Abcam, ab110641). pAb anti-CTCF (Diagenode, C15410210-50) was used as IP loading control. Following incubation with appropriate secondary antibodies, protein bands were revealed by the Pierce ECL Plus Western Blotting Substrate (ThermoScientific).

### 4.6. ChIP-seq Data Analysis

ChIP-seq data used in this study are from previous publications and are listed in Table S4 (proteins) and Table S5 (histone modifications) in Supporting Materials [29,52]. The LiftOver tool, available at the University of California Santa Cruz (UCSC) Genome Browser (https://genome.ucsc.edu/), was used when necessary to convert the genome coordinates from NCBI36/hg18 to GRCh37/hg19. After that, all the analyses were carried out using the GRCh37/hg19 coordinates. Data analyses were performed in R (3.6), importing the original bed files of peaks as GRangesList. First, for each protein/histone modification for which replicated tracks were available, we obtained consensus regions in bed format. The consensus regions were defined in terms of colocalizations between the replicates using the intersect function, i.e., taking as consensus peak coordinates the genomic regions common to all the replicates. When only one replicate was available, the consensus regions were identical to the peaks available for the sample. We removed regions smaller than 5 bp from the consensus lists, and we used the consensus peaks for the analyses. The number of consensus peaks for each histone marker and proteins in K562 cells is reported in Table S6A,B, respectively, while the number of consensus peaks for proteins in the HeLa-S3 cell line is reported in Table S6C.

Then, we computed the number of peaks for each factor that overlapped CTCF peaks using the countOverlaps function with default parameters and we obtained the corresponding lists of overlapping peaks. In each list, the peaks were reported uniquely, regardless of the number of overlaps in the genomic coordinate of the respective consensus list. Such analysis was performed independently in both HeLa-S3 and K562 cell lines. The number of peaks for each factor overlapping CTCF in the K562 and HeLa-S3 cell lines is reported in Table S7.

For the K562 cell line, we used the consensus peaks of the histone modifications to define the following genomic markers: active promoters (genomic regions with H3K4me3 peaks), inactive enhancers (genomic regions with H3K9me3 peaks), Inactive promoters (genomic regions with H3K27me3 peaks), active enhancers (the common genomic regions between H3K27ac and H3K4me1 peaks), and gene body (the common genomic regions between H3K36me3 and H3K79me2 peaks). The number of regions for active enhancers, active promoters, gene body, inactive enhancers, and inactive promoters for the K562 cell line is reported in Table S8A.

Then, we computed the overlap between the peaks of the core SMARCA4, ARID2, BRD9, and DPF2, that were overlapping CTCF, and the genomic coordinates. The number of protein peaks overlapping CTCF and the histone markers are reported in Table S8B.

### 4.7. Chemical Synthesis of the BRK Domain of Human BRG1

The sequence of the BRK domain of BRG1 (Accession number P51532), region 612–656, named hereafter BRK-BRG1, is MSDLPVKVIHVESGKILTGTDAPKAGQLEAWLEMNPGYEVAPRSD. The corresponding synthetic polypeptide was obtained by solid phase synthesis as the C-terminally amidated and N-terminally acetylated variant following the Fmoc/Tbu methodology [53,54], using an MBHA Rink amide (0.8 mmol/g) resin, on a synthesis scale of 76 µmol. Standard Fmoc protected amino acids (5-fold excess) were sequentially incorporated (RT, 30 min) using HATU/DIEA as coupling agent and piperidine 40% *v/v* in DMF for all deprotection steps (RT, 10 min). The final acetylation was performed using 30% *v*/*v* acetic anhydride in DMF containing 5% *v*/*v* DIEA for 30 min. The resin was finally washed and dried under vacuum. The polypeptide was cleaved from the solid support by treatment with a mixture of TFA/TIS/H_2_O 90/5/5 (*v*/*v*/*v*) for 3 h, at RT. The crude material was isolated by precipitation with cold diethyl ether and lyophilized. Purification was performed on a Jupiter C18 column (150 × 21.2 mm ID, 5 µm, Phenomenex, CA, United States) applying a gradient from 10% to 85% solvent B (CH_3_CN, 0.1% TFA) over solvent A (H_2_O, 0.1% TFA), for 20 min at a flow rate of 12 mL/min. Detection was at 214 nm. The purified fractions were analyzed by LC-MS ESI-TOF using a system composed by a 6230 ESI-TOF mass spectrometer (Agilent, Santa Clara, CA, USA) coupled to a 1290 Infinity LC System. An Onyx C18 column (50 × 2.1 mm ID, 5 µm, Phenomenex, Torrance, CA, USA) was used applying a gradient from 1% solvent B (CH_3_CN, 0.05% TFA) to 85% in 10 min at a flow rate of 0.2 mL/min. Solvent A was H_2_O, 0.05% TFA.

### 4.8. Recombinant CTCF Zinc Finger Domains 1–11 and 4–8 Expression and Purification

The plasmids CTCF-ZF1-11-pMalC2G and CTF-ZF4-8-pMalC2G, obtained as previously reported [39], were used to transform *E. coli* BL21 host strain. Transformed colonies were inoculated in LB 0.2% glucose medium and grown a 37 °C, until the culture reached the optical density of 0.6 nm at 600 nm (OD600). At this point of growth, 200 µM ZnSO_4_ was added to the culture and the expression of the protein was induced by using IPTG 0.3 mM. When the culture reached 0.9 OD600, bacterial cells were harvested by centrifugation. Pellets were resuspended in PBS (pH 7.4) and lysed by sonication on ice. Lysates were centrifuged for 30 min at 27.500 relative centrifugal force and the supernatants containing the expressed proteins were loaded on amylose resin (New England Biolabs, Milan, Italy), according to the manufacturer’s protocol, and incubated overnight under rotation. Following washes with 1X PBS, the purified proteins MBP-CTF-ZF4-8 and MBP-CTCF-ZF1-11 were obtained adding maltose elution buffer (10 mM maltose, 1X PBS). Then, the affinity-purified proteins were loaded onto an HiLoad 26/60 Superdex 75 gel filtration chromatography column (GE Healthcare, Milan, Italy) equilibrated and eluted with PBS. The purified products were finally concentrated using a Amicon Ultra Centrifugal Filter (Merck).

### 4.9. Binding of BRK-BRG1 to Recombinant CTFC Multiple Zinc Finger Domains by Biolayer Interferometry (BLI)

BLI measurements were performed using a BLItz system and ARG2 commercial biosensors (ForteBio, CA, United States), following the manufacturer’s instructions. A reduced volume sample cuvette (4 µL) was used for all the experiments. Assays were performed by immobilizing the synthetic BRK-BRG1 domain on the surface of the sensor chip and exposing it to solutions of recombinant CTFC domain 1–11 or CTCF domains 4–8, both fused to MBP, dissolved in PBS at increasing concentrations. After pre-hydration for 10 min in PBS buffer, the immobilization was achieved by pre-activating the sensor surface for 180 s with EDC/NHS, both in water, at 0.4 M and 0.1 M, respectively. Then, the sensor chip was exposed for 300 s to a solution of BRK-BRG1 at 200 µg/mL in sodium acetate pH 4.5. After extensive washing with buffer, the sensor chip surface was deactivated using 1 M ethanolamine in water pH 8.5 for 180 s. Binding assays were performed after exposure for 30 s to PBS (running buffer) to acquire the initial baseline. Next, the derivatized chip was exposed to the CTFC protein solutions in PBS for 120 s, followed by PBS washes (120 s). Then, the sensor chip was fully regenerated, restoring the baseline, by treatment with a solution of NaOH 5 mM for 10 s and a further wash with PBS.

The binding experiments with the MBP-fused CTFC domains 1–11 were performed at 0.5, 1.0, 1.5, 2.0, 2.5, and 3.0 µM. No shifts were measured with solutions at concentrations below 0.5 µM. For concentrations higher than 3.0 µM, the protein underwent precipitation preventing further tests. The binding experiments with the MBP-fused CTFC domain 4–8 were performed at 10, 31.5, 62.5, 100, 150, and 200 nM. No shifts were measured with solutions at concentrations below 10 nM. Control blank experiments were performed using free MBP at the same concentrations under the same experimental conditions. The shaker speed was set at 2000 rpm, according to the manufacturer’s instructions. Final interferograms were obtained by subtraction of the corresponding signals obtained with free MBP at the same concentrations. Data were exported from the BLItz Pro 1.2 software and replotted with GraphPad Prism, v. 5.00, GraphPad Software (San Diego, CA, USA). Plateau values (red dotted line) of binding, as reflected by changes in optical thickness (nm) at 150 s, were used to calculate the affinity constant (K_D_) by a nonlinear curve fitting and applying the one binding site hyperbola as model (GraphPad Prism).

## 5. Conclusions

Despite the amounts of information coming from genome-wide studies, to the best of our knowledge, this is the first evidence of a physical interaction between CTCF and SWI/SNF proteins. Although the biological significance of this interaction remains to be established, we believe that the inclusion of SWI/SNF members in the CTCF interaction network expands our knowledge of the protein–protein interaction landscape of this versatile transcription factor, highlighting an interplay between SWI/SNF and CTCF in several, perhaps intertwined processes related to genome topology and transcriptional regulation.

## Figures and Tables

**Table 1:** List of high-confidence members of the SWI/SNF remodeling complex identified by nano-LC-MS/MS. Mean number (#) of peptides and peptide-to-spectra matches (PSM) identified in replicate injections are reported. Alternative protein/gene names of identified subunits belonging to specific SWI/SNF complexes are reported in Figure 2. The asterisk (*) indicates a single peptide-based identification in both Benzonase-treated and untreated samples.

			Benzonase		Untreated		
Accession	Description	Name	# Peptides	# PSMs	# Peptides	# PSMs	MW (kDa)
**P51532**	Transcription activator BRG1	BRG1	30	47	29	48	185
**Q92922**	SWI/SNF complex subunit SMARCC1	BAF155	19	36	21	29	123
**Q8TAQ2**	SWI/SNF complex subunit SMARCC2	BAF170	16	25	15	19	133
**Q969G3**	SWI/SNF-related matrix-associated actin-dependent regulator of chromatin subfamily E member 1	BAF57	10	17	10	17	47
**Q96GM5**	SWI/SNF-related matrix-associated actin-dependent regulator of chromatin subfamily D member 1	BAF60A	9	13	10	15	58
**Q12824**	SWI/SNF-related matrix-associated actin-dependent regulator of chromatin subfamily B member 1	BAF47	6	9	9	11	44
**O14497**	AT-rich interactive domain-containing protein 1A	BAF250A	33	57	29	50	242
**Q92785**	Zinc finger protein ubi-d4	BAF45D	10	15	10	15	44
**Q8NFD5**	AT-rich interactive domain-containing protein 1B	BAF250B	2	2	3	3	236
**Q9H8M2**	Bromodomain-containing protein 9	BRD9	1	1	3	3	67
**Q9NPI1**	Bromodomain-containing protein 7 *	BRD7	1	4	1	3	74
**P60709**	Actin, cytoplasmic 1	ACTB	19	78	12	33	42
**O96019**	Actin-like protein 6A	BAF53A	4	7	5	7	47
**Q15532**	Protein SSXT	SS18	2	4	2	3	46

## Supplementary Materials

The following are available online at https://www.mdpi.com/1422-0067/21/23/8950/s1.

## Abbreviations

AP-MS	Affinity-purification mass spectrometry
BAF	BRG1/BRM associated factor
CTCF	CCCTC-binding factor
CHD	Chromodomain helicase DNA-binding
IP	Immunoprecipitation
ncBAF	Non-canonical BAF
PBAF	Polybromo-associated BAF
PSM	Peptide-to-spectra matches
SWI/SNF	Switch/sucrose nonfermentable
TADs	Topological associated domains
