# Supplementary material for: Exploring the Interaction between the SWI/SNF Chromatin Remodeling Complex and the Zinc Finger Factor CTCF
Source: Int J Mol Sci. 2020 Nov 25;21(23):8950. doi: 10.3390/ijms21238950 (PMC7728349; doi:10.3390/ijms21238950)

Supporting Information

Exploring the interaction between the SWI/SNF chromatin remodeling complex and the zinc finger factor CTCF

Mariangela Valletta[1#], Rosita Russo[1#], Ilaria Baglivo[1], Veronica Russo[1], Sara Ragucci[1], Annamaria Sandomenico[2], Emanuela Iaccarino[2], Menotti Ruvo[2], Italia De Feis[3], Claudia Angelini[3], Sara Iachettini[4], Annamaria Biroccio[4], Paolo Vincenzo Pedone[1*] and Angela Chambery[1*]

[1]Department of Environmental, Biological and Pharmaceutical Sciences and Technologies, University of Campania "Luigi Vanvitelli", 81100 Caserta, Italy
[2] Istituto di Biostrutture e Bioimmagini IBB, National Research Council, Via Mezzocannone 16, 80134 Napoli, Italy
[3]Istituto per le applicazioni del calcolo IAC 'M. Picone', National Research Council, Via Pietro Castellino 111, 80131, Napoli, Italy
[4] Oncogenomic and Epigenetic Unit, IRCCS-Regina Elena National Cancer Institute, Via Elio Chianesi 53, 00144 Roma, Italy

Running title: *CTCF interacts with the SWI/SNF complex*

[*]To whom correspondence should be addressed: Paolo Vincenzo Pedone and Angela Chambery: Department of Environmental, Biological and Pharmaceutical Sciences and Technologies, University of Campania "Luigi Vanvitelli", 81100 Caserta, Italy;
paolovincenzo.pedone@unicampania.it; Tel. (+39) 0823275150
angela.chambery@unicampania.it; Tel. (+39) 0823274583

[#]M.V. and R.R. contributed equally to this work.

Figure S1. Bubble plot showing a clusterization of identified proteins according to the three classes of mammalian SWI/SNF complexes: canonical BRG1/BRM-associated factor (BAF), polybromo-associated BAF (PBAF) and ncBAF complexes (30). Bubble size is related to number of peptides identified by MS indicated within the bubbles.

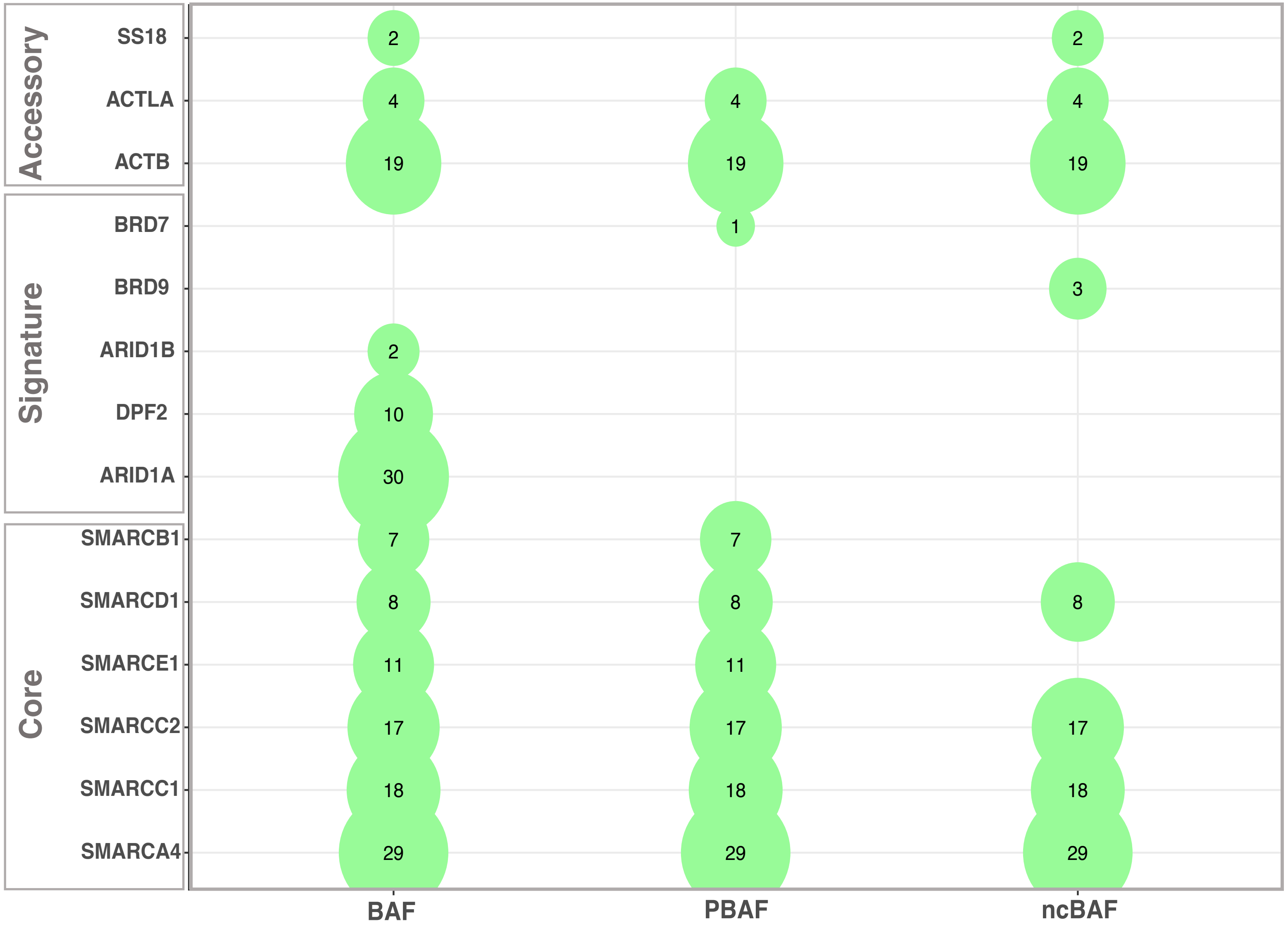

Figure S2. A) Scatter plot showing the distribution of peptide-to-spectra matches (PSM) mapped on BRG1 according to their monoisotopic masses (m/z) vs. the retention times (min). The colour of the spots indicate the accuracy between theoretical and experimental molecular masses according to the binary comparison plot reported in B along with the colour gradient bar of Δ(ppm) values. C) Representative base peak intensity chromatogram obtained from the high-resolution LC-MS DDA analysis of the tryptic digest of the CTCF IP sample treated with Benzonase showing in red the retention times of peptide-to-spectra matches (PSM) used for identification of BRG1.

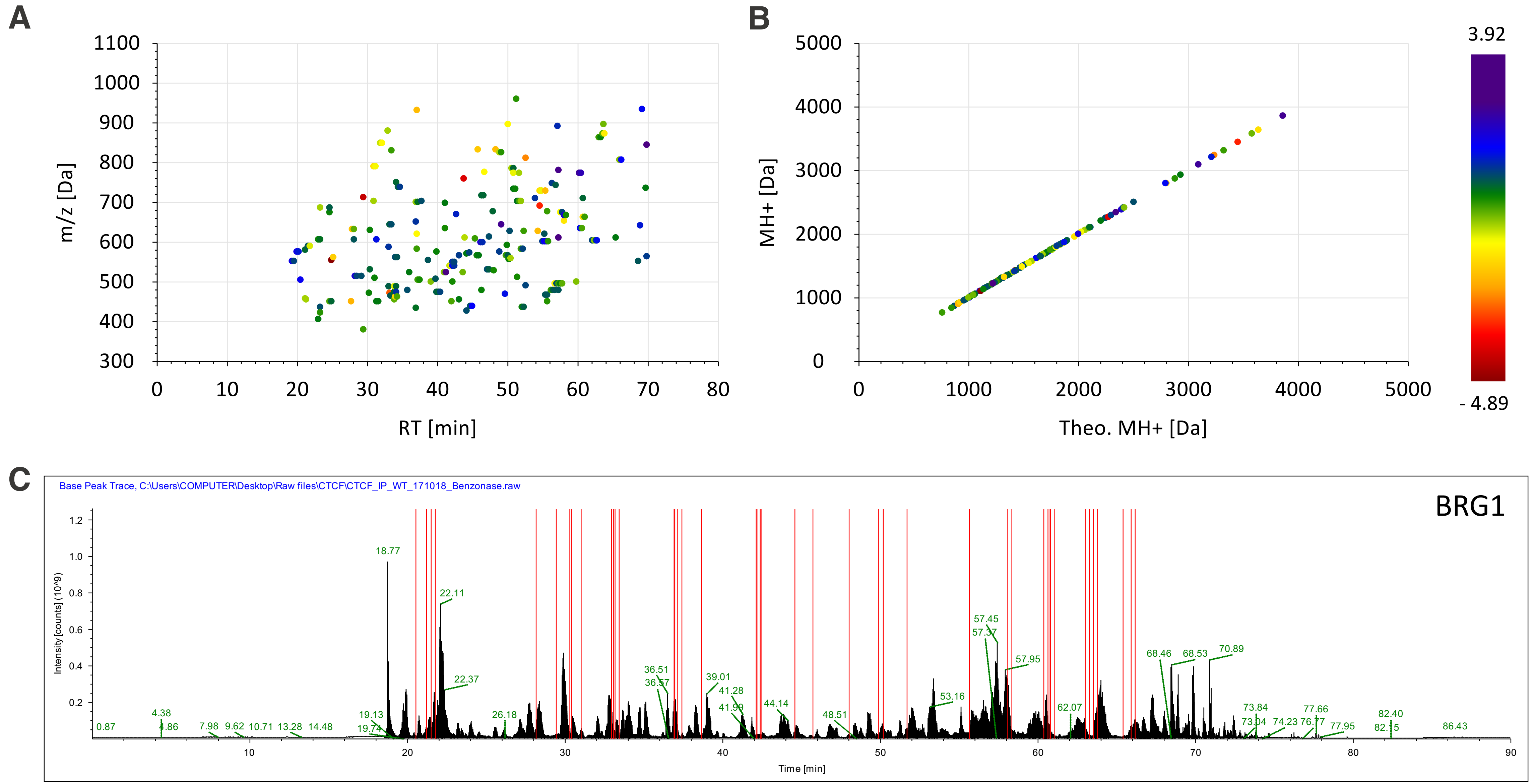

Figure S3. Representative base peak intensity (BPI) chromatograms obtained from the high-resolution LC-MS DDA analysis of the tryptic digest of the CTCF IP sample treated with Benzonase showing in red the retention times of peptide-to-spectra matches (PSM) used for the identification of BAF155 (A) and BAF170 (B).

A

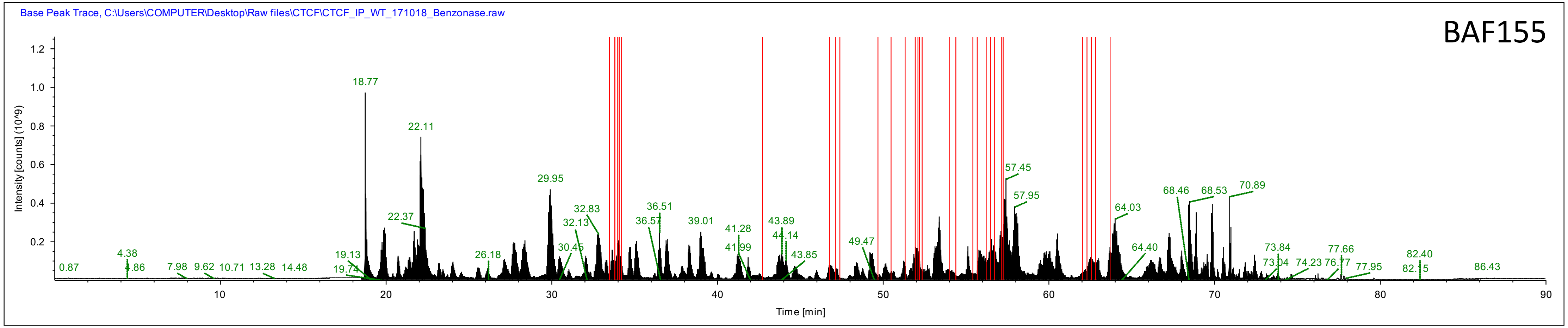

B

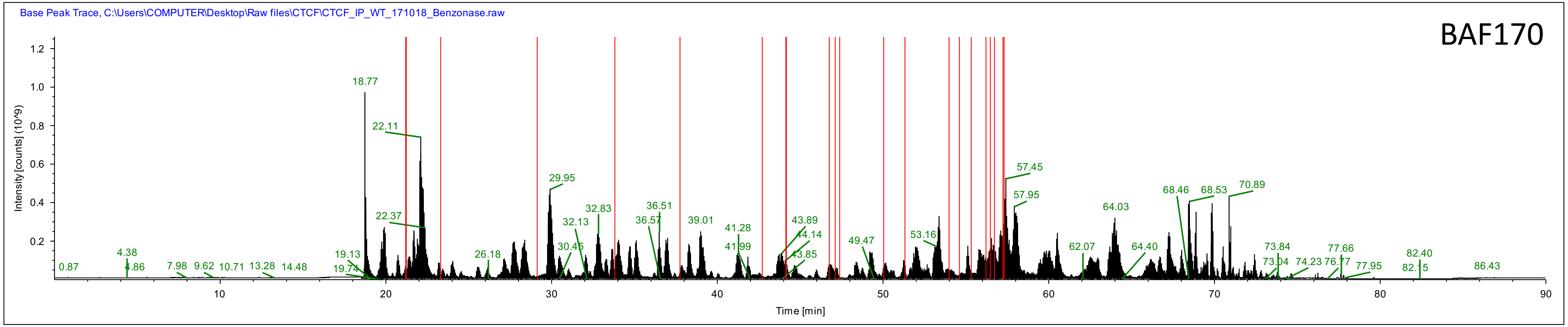

Figure S4. Representative MS/MS spectra of peptides mapping on BAF155. A) Doubly charged ion at m/z 776.874 corresponding to the sequence region 500-512, cabamidomethylated (57.021 Da) on C511 (LNPQEYLTSTACR; theoretical MH+: 1552.742 Da; Δ(ppm)=-1.14; XCorr=2.3); B) Doubly charged ion at m/z 890.432 corresponding to the sequence region 42-56 (FWESPETVSQLDSVR; theoretical MH+: 1779.855 Da; Δ(ppm)=1.09;XCorr=2.9).

A

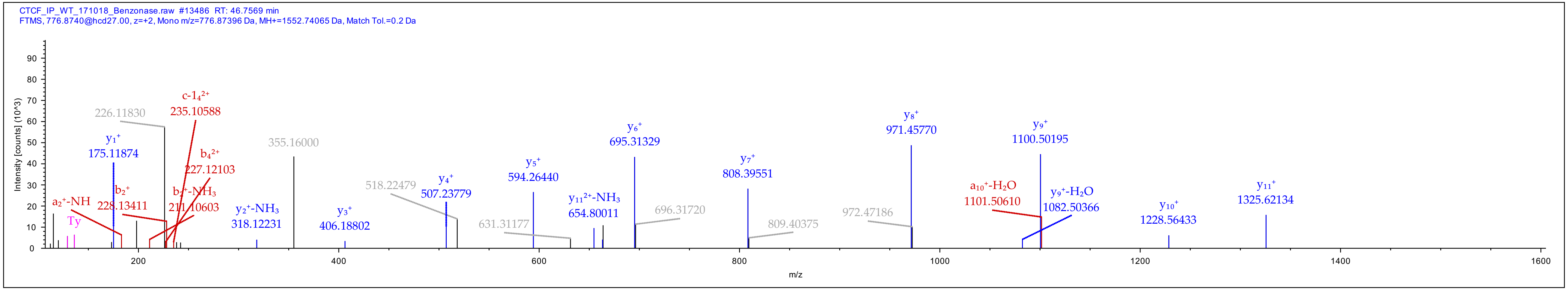

B

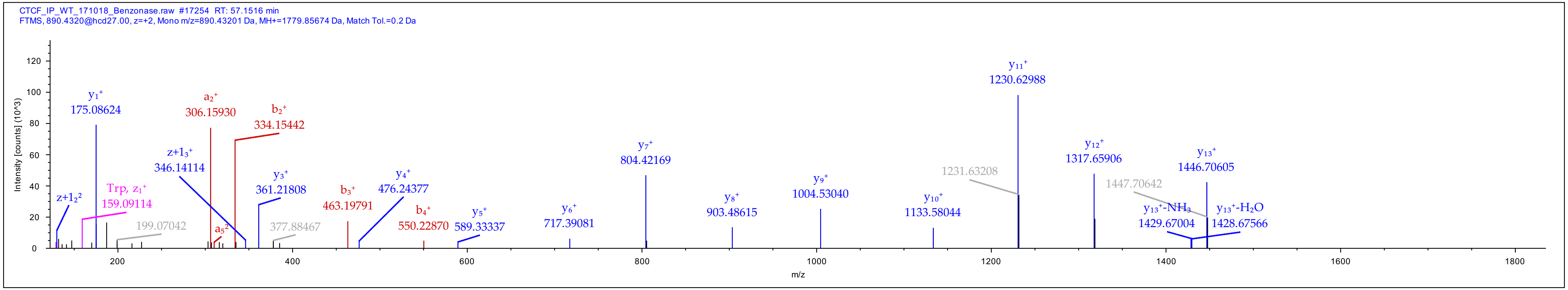

Figure S5. Representative MS/MS spectra of peptides mapping on BAF170. A) Doubly charged ion at m/z 896.413 corresponding to the sequence region 14-28 (YYEAADTVTQFDNVR, theoretical MH+: 1791.818 Da; Δ(ppm)=-1.16; XCorr=3.2); B) Triply charged ion at m/z 570.307 corresponding to the sequence region 703-717 (MKEEVPTALVEAHVR; theoretical MH+: 1708.905 Da; Δ(ppm)=0.28; XCorr=3.1).

A

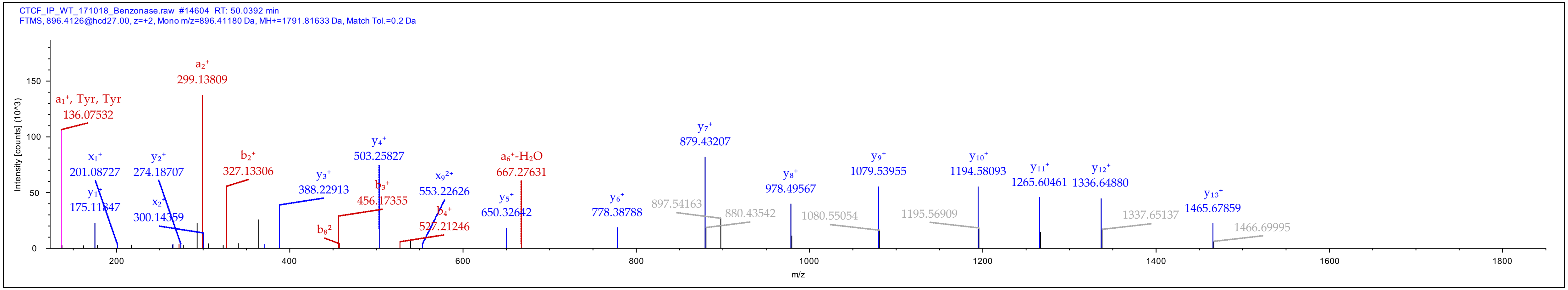

B

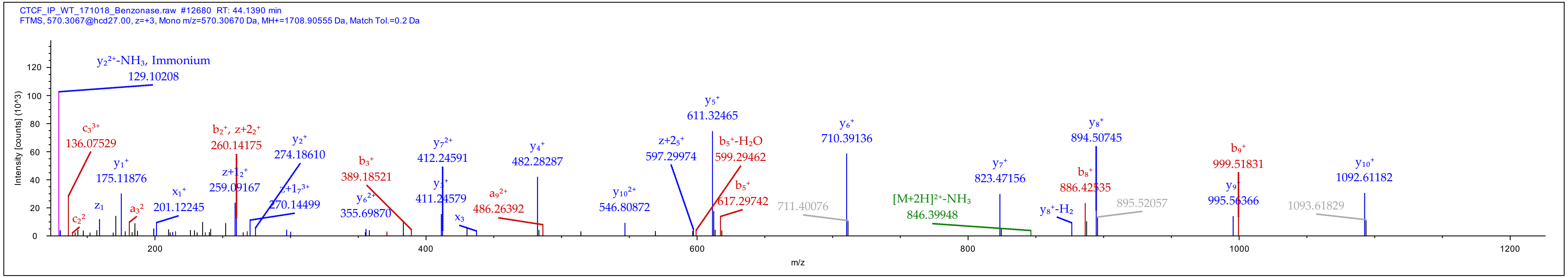

Figure S6. Representative MS/MS spectra of peptides mapping on BRG1 and BAF57 (A-B and C-D, respectively). A) Triply charged ion at m/z 527.611 corresponding to the sequence region 472-484 (HQEYLNSILQHAK; theoretical MH^+: 1580.818 Da; Δ(ppm)=0.12; XCorr=3.7); B) Doubly charged ion at m/z 651.854 corresponding to the sequence region 426-437 (RDTALETALNAK; theoretical MH^+: 1302.701 Da; Δ(ppm)=0.95; XCorr=1.7). C) Doubly charged ion at m/z 700.362 corresponding to the sequence region 285-297 (IAAEIAQAEEQAR; theoretical MH^+: 1399.717 Da; Δ(ppm)=-0.53; XCorr=2.5). D) Triply charged ion at m/z 565.955 corresponding to the sequence region 132-146 (AYHNSPAYLAYINAK, theoretical MH^+: 1695.850; Δ(ppm)=0.49; XCorr=3.5).

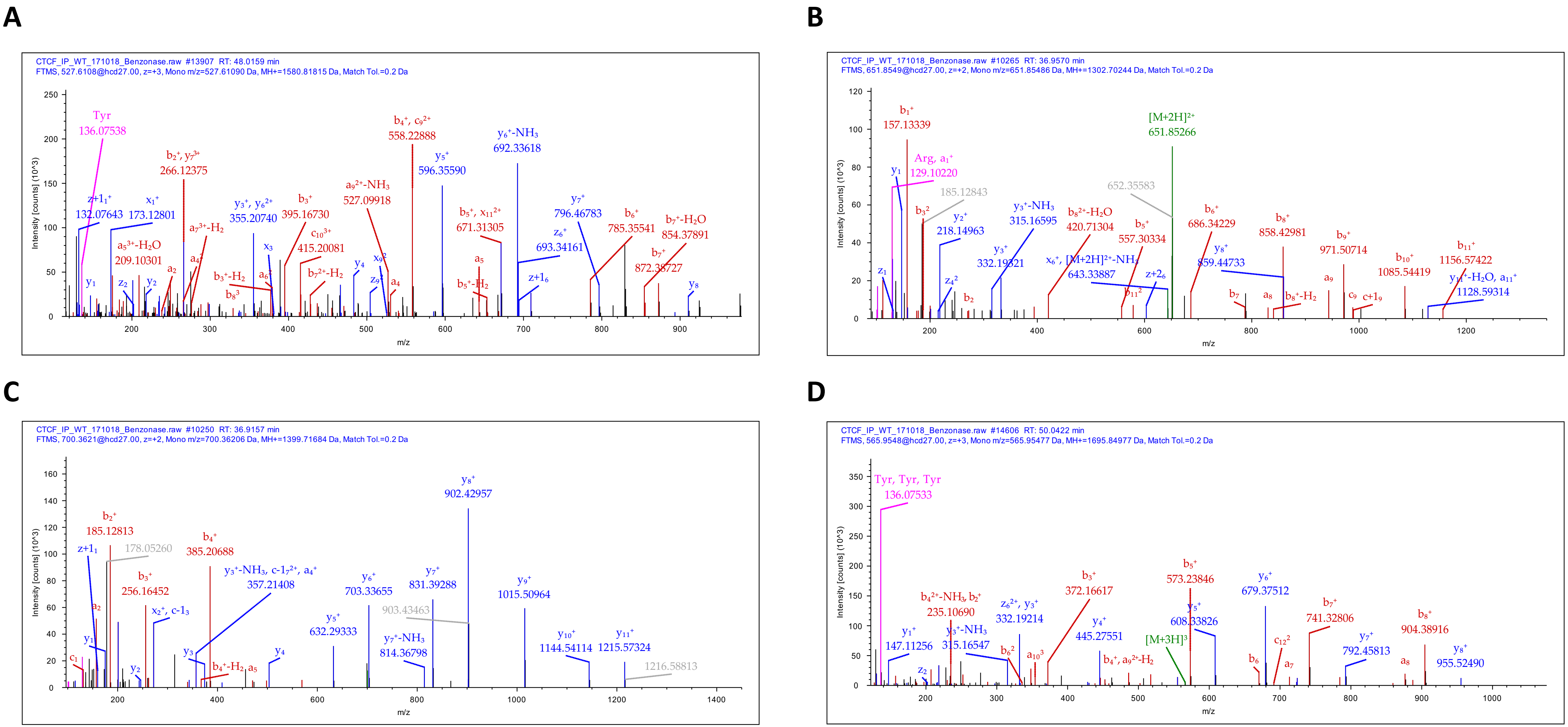

Figure S7. Representative MS/MS spectra of peptides mapping on BAF60A and BAF47 (A-B and C-D, respectively). A) Doubly charged ion at m/z 738.836 corresponding to the sequence region 465-477 (TMTDVVGNPEEER; theoretical MH$^+$: 1476.665 Da; Δ(ppm)=0.91; XCorr=1.7); B) Triply charged ion at m/z 438.250 corresponding to the sequence region 403-413 (RQELEQALGIR; theoretical MH$^+$: 1312.734 Da; Δ(ppm)=0.69; XCorr=3.7). C) Doubly charged ion at m/z 628.274 corresponding to the sequence region 202-211 (DAFTWNMNEK; theoretical MH$^+$: 1255.541 Da; Δ(ppm)=-0.11; XCorr=2.0). D) Doubly charged ion at m/z 774.356 corresponding to the sequence region 93-106 (ASEVEEILDGNDEK, theoretical MH$^+$: 1547.706; Δ(ppm)=-0.98; XCorr=3.1).

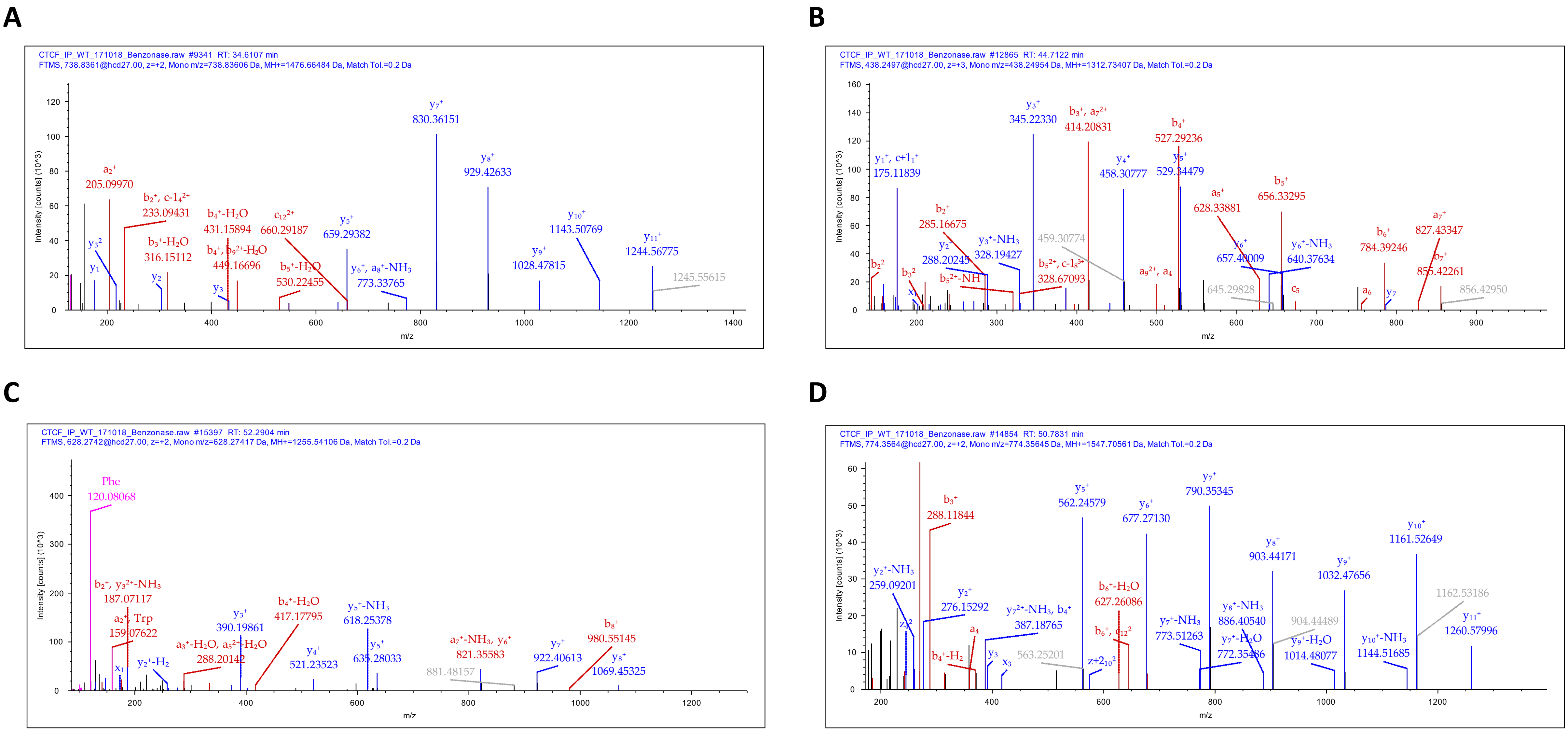

Figure S8. Representative MS/MS spectra of peptides mapping on BAF250A and BAF45D (A-B and C-D, respectively). A) Triply charged ion at m/z 575.929 corresponding to the sequence region 2244-2257 (VDENHSEFTLYESR; theoretical MH$^+$: 1725.772 Da; Δ(ppm)= 1.34; XCorr=3.3); B) Triply charged ion at m/z 576.280 corresponding to the sequence region 1512-1528 (QSTGSAPQGPAYHGVNR; theoretical MH$^+$: 1726.828 Da; Δ(ppm)= 0.3; XCorr=4.7). C) Doubly charged ion at m/z 825.884 corresponding to the sequence region 138-152 (VDDDSLGEFPVTNSR; theoretical MH$^+$: 1650.760 Da; Δ(ppm)=-0.01; XCorr=3.0). D) Doubly charged ion at m/z 844.449 corresponding to the sequence region 109-124 (VDDDSLGEFPVTNSR, theoretical MH$^+$: 1687.891; Δ(ppm)=2.92; XCorr=3.0).

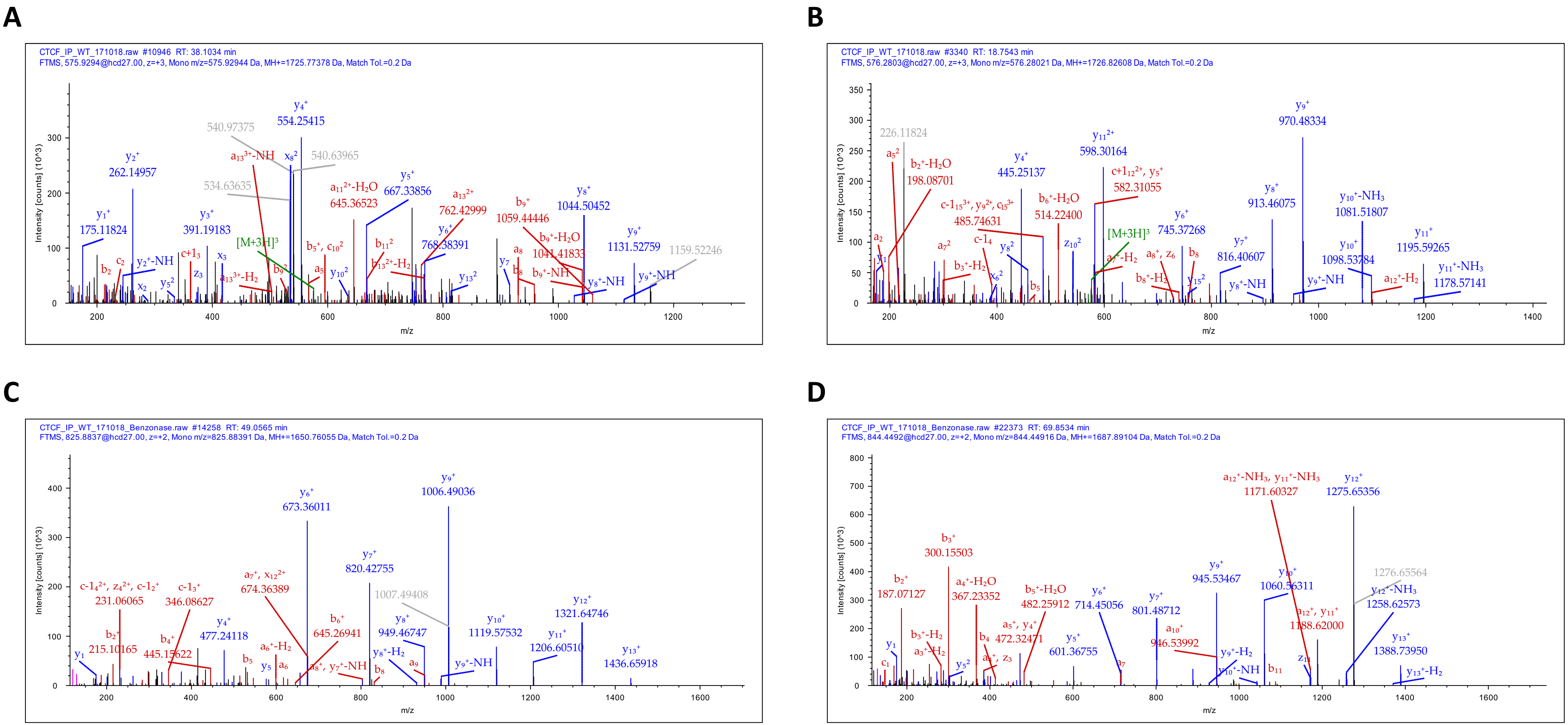

Figure S9. Representative MS/MS spectra of peptides mapping on BAF250B and BRD9 (A-B and C-D, respectively). A) Quadruply charged ion at m/z 574.778 corresponding to the sequence region 1218-1238 (SSTISVHDPFSDVSDSSFPKR; theoretical MH$^+$: 2295.091 Da; Δ(ppm)=0.84; XCorr=2.4); B) Doubly charged ion at m/z 436.230 corresponding to the sequence region 1905-1911 (LLEDEPR; theoretical MH$^+$: 871.453 Da; Δ(ppm)=0.75; XCorr=1.5). C) Triply charged ion at m/z 615.288 corresponding to the sequence region 514-529 (ELDPDDSHLNLDETTK; theoretical MH$^+$: 1841.842 Da; Δ(ppm)=1.29; XCorr=2.6). D) Triply charged ion at m/z 623.657 corresponding to the sequence region 438-454 (KVVDDLLDQITGGDHSR, theoretical MH$^+$: 1867.952; Δ(ppm)=0.56; ; XCorr=2.4).

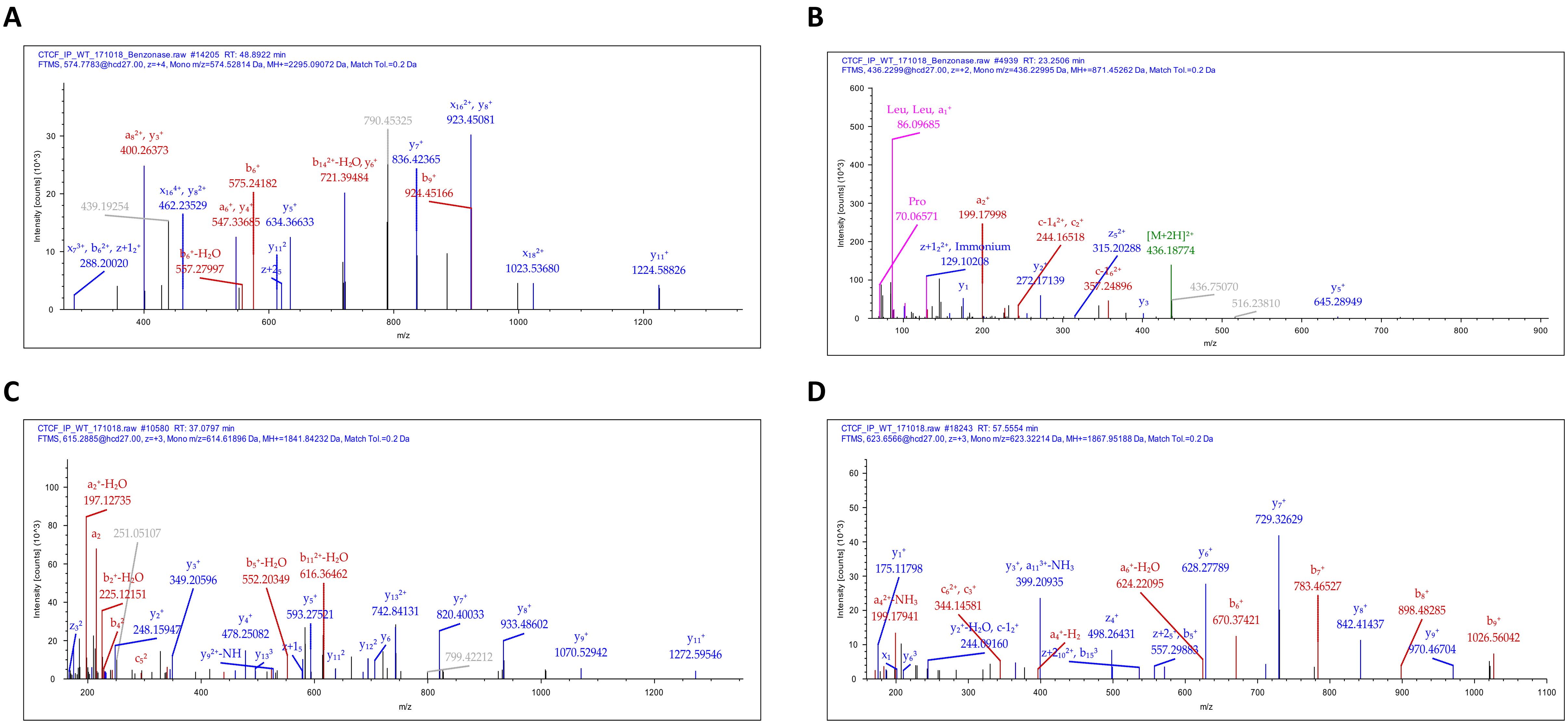

Figure S10. Representative MS/MS spectra of peptides mapping on BAF53A and ACTB (A-B and C-D, respectively). A) Doubly charged ion at m/z 784.889 corresponding to the sequence region 63-76 (QGGPTYYIDTNALR; theoretical MH^+: 1568.771 Da; Δ(ppm)=0.37; XCorr=3.1); B) Triply charged ion at m/z 676.002 corresponding to the sequence region 113-130 (SEASLHPVLMSEAPWNTR; theoretical MH^+: 2024.986 Da; Δ(ppm)=0.11; XCorr=4.3). C) Doubly charged ion at m/z 586.289 corresponding to the sequence region 40-50 (HQGVMVGMGQK; theoretical MH^+: 1171.572 Da; Δ(ppm)=0.55; XCorr=3.5). D) Doubly charged ion at m/z 581.313 corresponding to the sequence region 316-326 (EITALAPSTMK, theoretical MH^+: 1161.619; Δ(ppm)=0.59; XCorr=2.4).

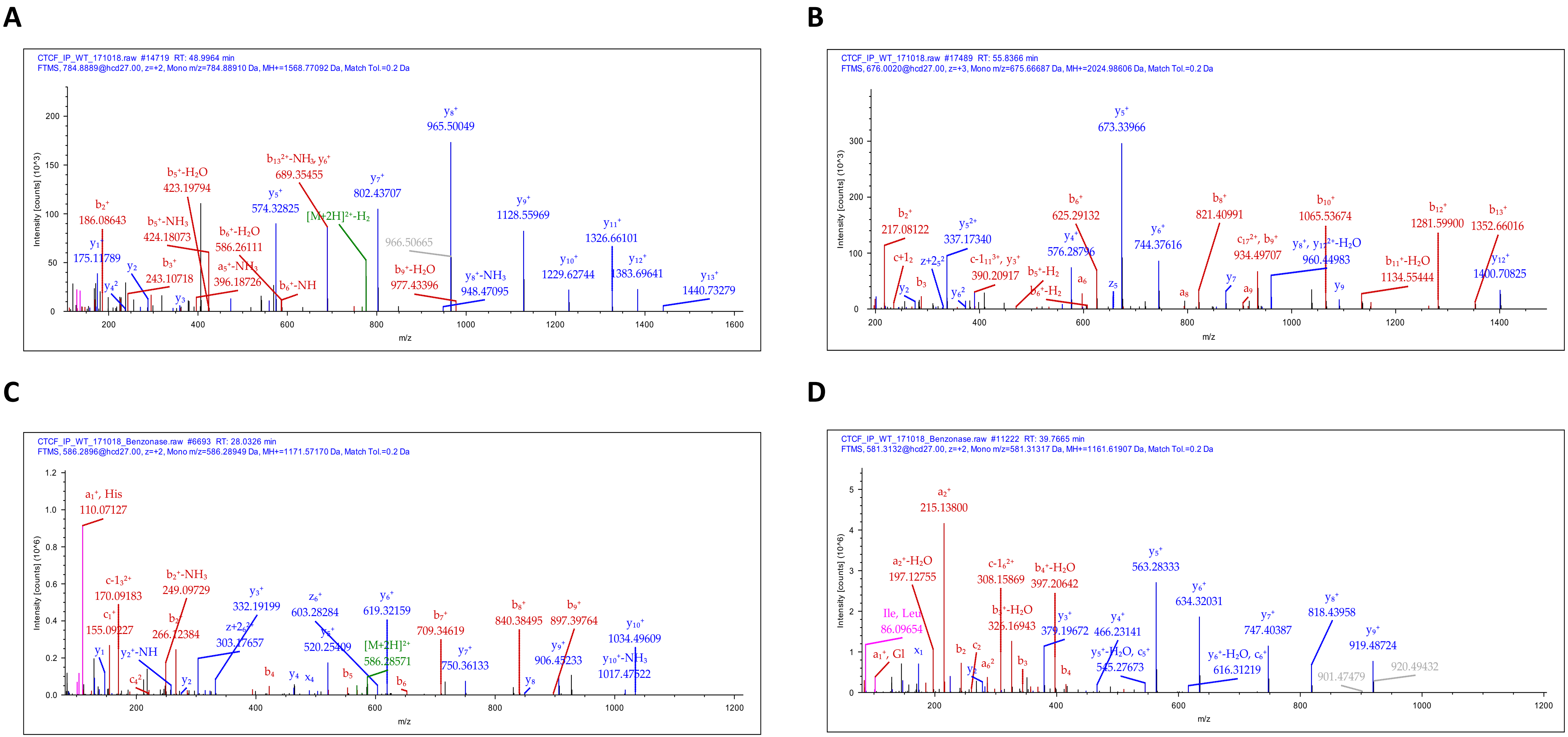

Figure S11. Representative MS/MS spectra of peptides mapping on SS18. A) Triply charged ion at m/z 730.330 corresponding to the sequence region 24-41, cabamidomethylated (57.021 Da) on C34 (MLDDNNHLIQCIMDSQNK; theoretical MH^+: 2188.977 Da; Δ(ppm)=-0.57; XCorr=2.9); B) Doubly charged ion at m/z 514.293 corresponding to the sequence region 14-23 (GEITPAAIQK; theoretical MH^+: 1027.579 Da; Δ(ppm)=0.77; XCorr=1.9).

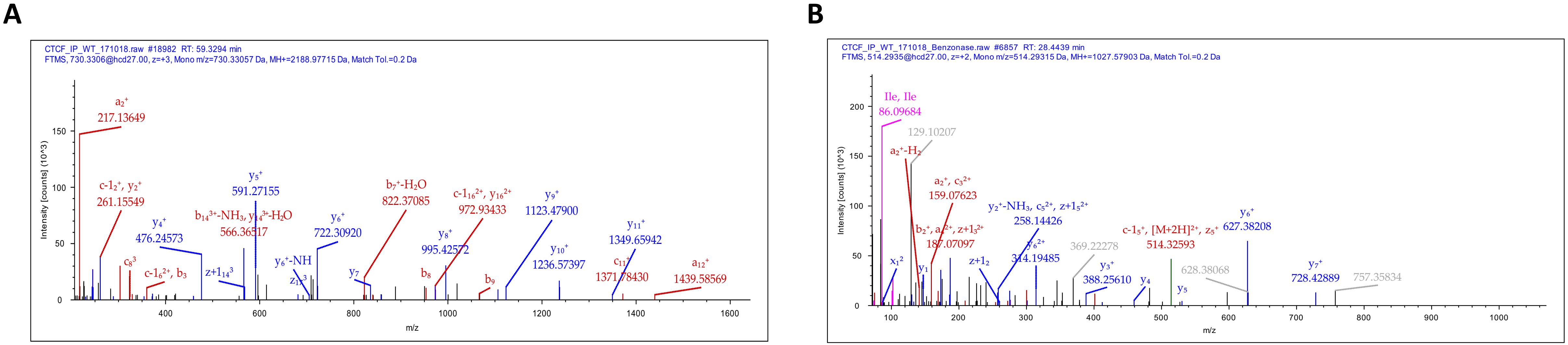

Figure S12. MS/MS spectra of the peptide mapping on BRD7 identified in benzonase-treated (A) and untreated (B) samples. A) Quintuply charged ion at m/z 729.348 corresponding to the sequence region 151-182, oxidized on M181 (DPSAFFSFPVTDFIAPGYSMIIKHPMDFSTMK; theoretical MH^+: 3640.720 Da; Δ(ppm)=-0.94; XCorr=2.4). B) Quintuply charged ion at m/z 729.348 corresponding to the sequence region 151-182, oxidized on M181 (DPSAFFSFPVTDFIAPGYSMIIKHPMDFSTMK; theoretical MH^+: 3640.720 Da; Δ(ppm)=1.16; XCorr=2.6).

A

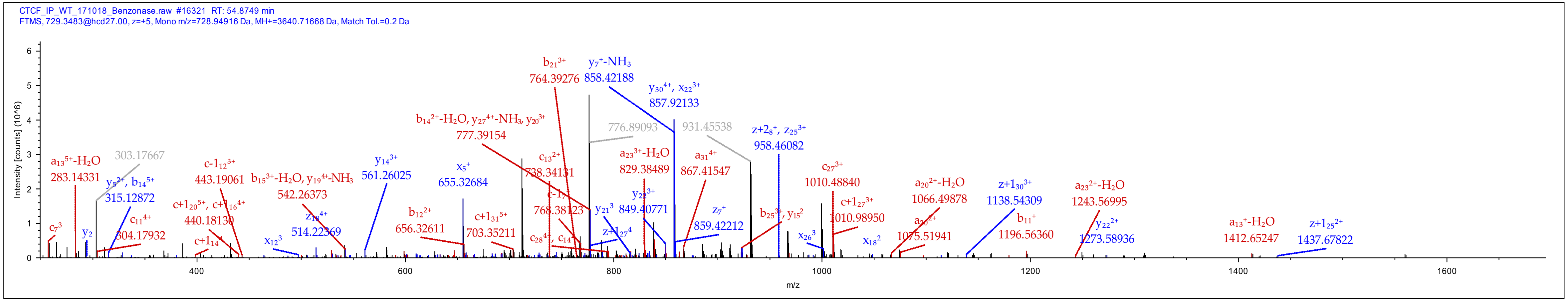

B

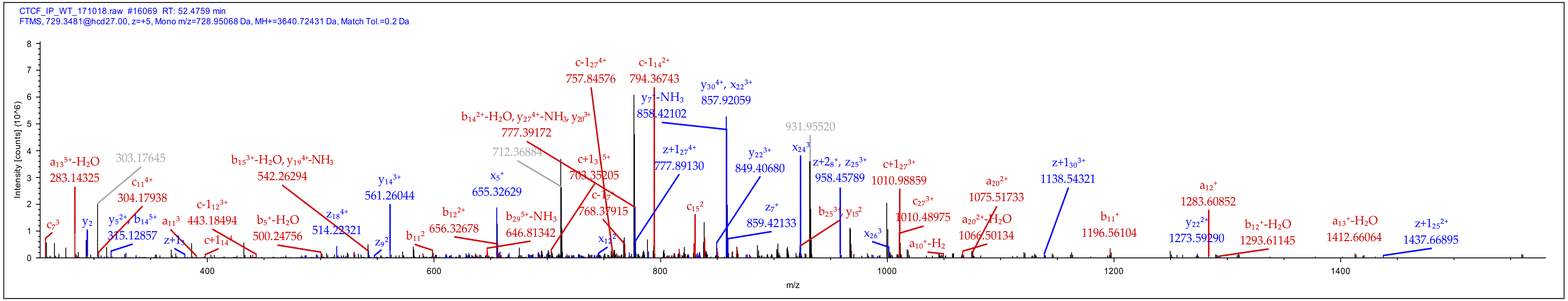

Figure S13. HPLC chromatogram referred to the synthetic purified BRK-BRG1. B. Deconvoluted mass spectrum of the product analysed in A. The experimental MW (4907.45 amu) is in agreement with the theoretical value of 4892.63 amu considering an oxidation on one of the two methionines (+ 16 amu) present in the BRK sequence (average; Δmass = 15.45 amu).

A

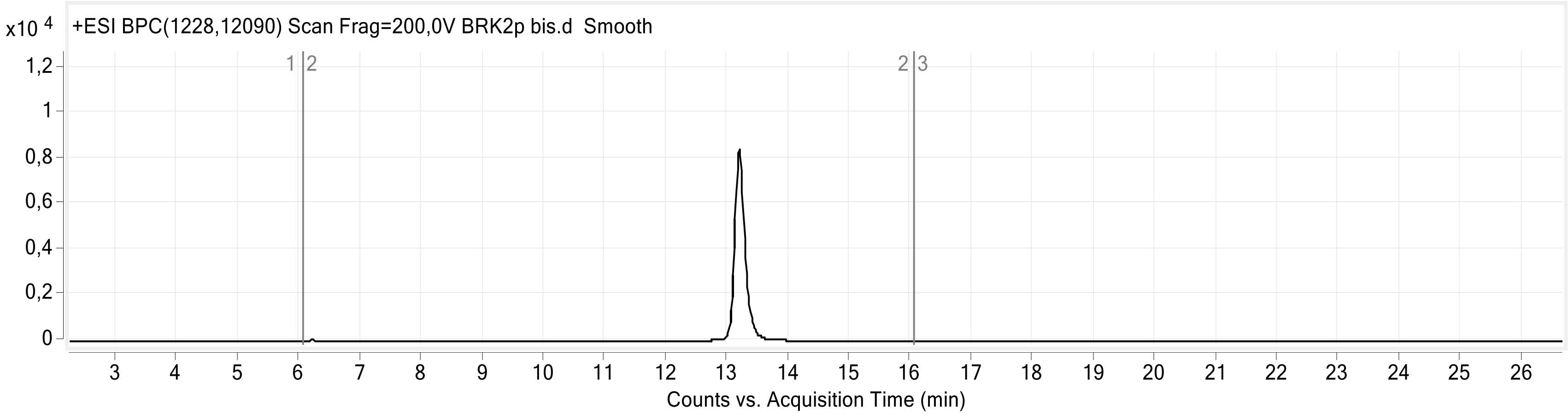

B

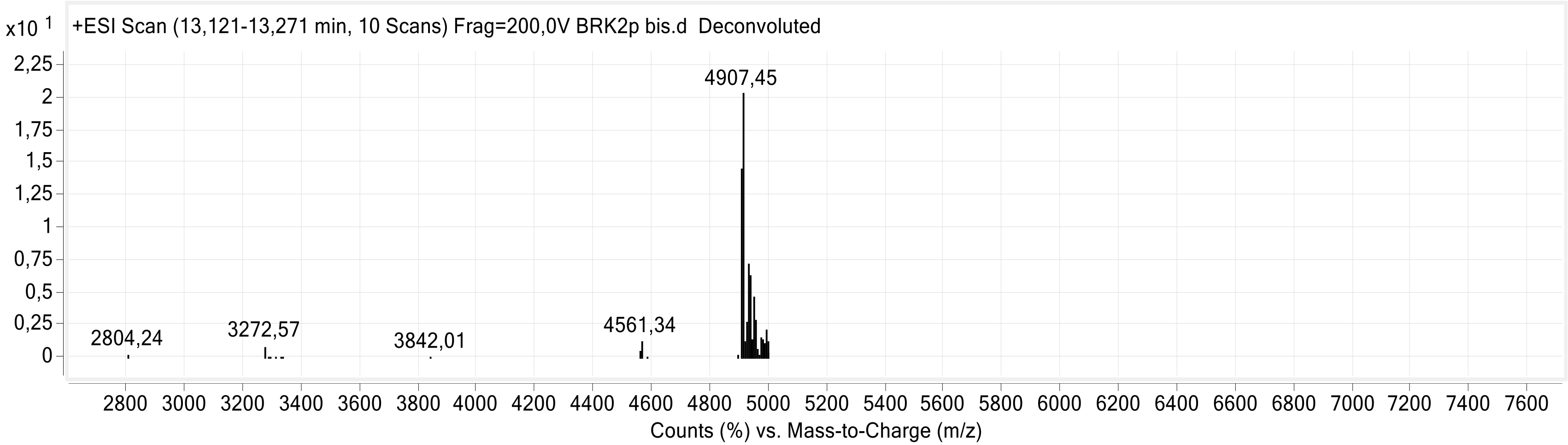

Supplement: Supplementary file 1 [file ijms-21-08950-s001.zip › Supplementary material_R2/SM_Figures S1-S13.pdf]
