# Supplementary material for: Exploring the Interaction between the SWI/SNF Chromatin Remodeling Complex and the Zinc Finger Factor CTCF
Source: Int J Mol Sci. 2020 Nov 25;21(23):8950. doi: 10.3390/ijms21238950 (PMC7728349; doi:10.3390/ijms21238950)
Supplement: Supplementary file 1 [file ijms-21-08950-s001.zip › Supplementary material_R2/SM_Tables S1-S8.pdf]

Supporting Information

Exploring the interaction between the SWI/SNF chromatin remodeling complex and the zinc finger factor CTCF

Mariangela Valletta[1#], Rosita Russo[1#], Ilaria Baglivo[1], Veronica Russo[1], Sara Ragucci[1], Annamaria Sandomenico[2], Emanuela Iaccarino[2], Menotti Ruvo[2], Italia De Feis[3], Claudia Angelini[3], Sara Iachettini[4], Annamaria Biroccio[4], Paolo Vincenzo Pedone[1*] and Angela Chambery[1*]

[1]Department of Environmental, Biological and Pharmaceutical Sciences and Technologies, University of Campania “Luigi Vanvitelli”, 81100 Caserta, Italy
[2] Istituto di Biostrutture e Bioimmagini IBB, National Research Council, Via Mezzocannone 16, 80134 Napoli, Italy
[3]Istituto per le applicazioni del calcolo IAC ‘M. Picone’, National Research Council, Via Pietro Castellino 111, 80131, Napoli, Italy
[4] Oncogenomic and Epigenetic Unit, IRCCS-Regina Elena National Cancer Institute, Via Elio Chianesi 53, 00144 Roma, Italy

Running title: *CTCF interacts with the SWI/SNF complex*

[*]To whom correspondence should be addressed: Paolo Vincenzo Pedone and Angela Chambery: Department of Environmental, Biological and Pharmaceutical Sciences and Technologies, University of Campania “Luigi Vanvitelli”, 81100 Caserta, Italy;
paolovincenzo.pedone@unicampania.it; Tel. (+39) 0823275150
angela.chambery@unicampania.it; Tel. (+39) 0823274583

[#]M.V. and R.R. contributed equally to this work.

Table S1. Identification details of the SWI/SNF remodeling complex subunits identified by nano-LC–MS/MS by searching the custom SWI/SNF database in triplicate injections of A) Benzonase-treated and B) untreated samples. PSM, peptide-to-spectra matches.

A

						Benzonase (inj #1)						Benzonase (inj #2)						Benzonase (inj #3)					
Accession	Description	Gene names	# AAs	MW [kDa]	calc. pI	Coverage	# Peptides	# PSMs	# Unique Peptides	Score Sequest HT	# Peptides Sequest HT	Coverage	# Peptides	# PSMs	# Unique Peptides	Score Sequest HT	# Peptides Sequest HT	Coverage	# Peptides	# PSMs	# Unique Peptides	Score Sequest HT	# Peptides Sequest HT
O14497	AT-rich interactive domain-containing protein 1A	BAF250A/ARID1A	2285	241.892	6.70	19	30	53	30	153	30	19	28	50	28	156	28	24	40	69	40	171	40
P51532	Transcription activator BRG1	BRG1/SMARCA4	1647	184.53	7.88	19	29	44	29	118	29	16	23	38	23	114	23	24	39	60	39	135	39
Q92922	SWI/SNF complex subunit SMARCC1	BAF155/SMARCC1	1105	122.79	5.76	21	18	32	12	87	18	22	18	33	13	97	18	20	21	44	14	102	21
Q8TAQ2	SWI/SNF complex subunit SMARCC2	BAF170/SMARCC2	1214	132.797	5.69	17	17	22	11	48	17	12	10	18	5	48	10	20	22	35	15	59	22
Q969G3	SWI/SNF-related matrix-associated actin-dependent regulator of chromatin subfamily E member 1	BAF57/SMARCE1	411	46.621	4.88	33	11	16	11	42	11	25	9	14	9	43	9	33	11	20	11	47	11
Q96GM5	SWI/SNF-related matrix-associated actin-dependent regulator of chromatin subfamily D member 1	BAF60A/SMARCD1	515	58.196	9.25	24	8	12	8	35	8	21	6	9	6	30	6	29	12	17	10	37	12
Q92785	Zinc finger protein ubi-d4	BAF45D/DPF2	391	44.127	6.33	46	10	13	10	34	10	37	8	11	8	34	8	49	13	22	13	34	13
Q12824	SWI/SNF-related matrix-associated actin-dependent regulator of chromatin subfamily B member 1	BAF47/SMARCB1	385	44.113	6.23	22	7	9	7	20	7	19	6	7	6	18	6	19	6	10	6	23	6
O96019	Actin-like protein 6A	BAF53A/ACTLA	429	47.43	5.60	17	4	7	4	20	4	17	4	6	4	20	4	17	4	7	4	20	4
Q15532	Protein SSXT	SS18	418	45.899	6.46	3	2	5	2	11	2	3	1	2	1	5	1	7	3	6	3	11	3
Q8NFD5	AT-rich interactive domain-containing protein 1B	BAF250B/ARID1B	2236	235.974	6.73	1	2	2	2	2	2	-	-	-	-	-	-	1	2	2	2	2	2
Q9NPI1	Bromodomain-containing protein 7	BRD7	651	74.092	6.39	5	1	4	1	0	1	-	-	-	-	-	-	-	-	-	-	-	-
P60709	Actin, cytoplasmic 1	β-actin/ACTB	375	41.71	5.48	54	19	75	19	203	19	50	16	63	16	191	16	54	21	96	21	225	21
Q9H8M2	Bromodomain-containing protein 9	BRD9	597	66.958	6.19	-	-	-	-	-	-	-	-	-	-	-	-	2	1	1	1	0	1

B

						Untreated (inj #1)						Untreated (inj #2)						Untreated (inj #3)					
Accession	Description	Gene names	# AAs	MW [kDa]	calc. pI	Coverage	# Peptides	# PSMs	# Unique Peptides	Score Sequest HT	# Peptides Sequest HT	Coverage	# Peptides	# PSMs	# Unique Peptides	Score Sequest HT	# Peptides Sequest HT	Coverage	# Peptides	# PSMs	# Unique Peptides	Score Sequest HT	# Peptides Sequest HT
O14497	AT-rich interactive domain-containing protein 1A	BAF250A/ARID1A	2285	241.892	6.70	17	24	43	24	136	24	18	27	47	27	145	27	21	37	61	36	161	37
P51532	Transcription activator BRG1	BRG1/SMARCA4	1647	184.53	7.88	18	24	36	24	108	24	19	26	42	26	125	26	22	38	65	38	149	38
Q92922	SWI/SNF complex subunit SMARCC1	BAF155/SMARCC1	1105	122.79	5.76	19	16	22	12	67	16	21	20	26	15	78	20	28	26	40	19	89	26
Q8TAQ2	SWI/SNF complex subunit SMARCC2	BAF170/SMARCC2	1214	132.797	5.69	13	10	12	6	34	10	16	13	16	8	43	13	21	22	30	15	48	22
Q969G3	SWI/SNF-related matrix-associated actin-dependent regulator of chromatin subfamily E member 1	BAF57/SMARCE1	411	46.621	4.88	21	8	13	8	40	8	21	10	17	10	49	10	29	13	21	13	55	13
Q96GM5	SWI/SNF-related matrix-associated actin-dependent regulator of chromatin subfamily D member 1	BAF60A/SMARCD1	515	58.196	9.25	14	7	10	7	34	7	14	7	11	7	36	7	27	16	23	12	44	16
Q92785	Zinc finger protein ubi-d4	BAF45D/DPF2	391	44.127	6.33	39	8	12	8	42	8	43	10	14	10	46	10	47	13	20	13	48	13
Q12824	SWI/SNF-related matrix-associated actin-dependent regulator of chromatin subfamily B member 1	BAF47/SMARCB1	385	44.113	6.23	29	7	9	7	27	7	34	9	11	9	32	9	34	10	14	10	34	10
O96019	Actin-like protein 6A	BAF53A/ACTLA	429	47.43	5.60	14	3	5	3	17	3	23	6	8	6	23	6	23	6	9	6	26	6
Q15532	Protein SSXT	SS18	418	45.899	6.46	7	2	2	2	6	2	7	2	2	2	6	2	7	3	4	3	9	3
Q8NFD5	AT-rich interactive domain-containing protein 1B	BAF250B/ARID1B	2236	235.974	6.73	-	-	-	-	-	-	-	-	-	-	-	-	2	3	3	2	5	3
Q9NPI1	Bromodomain-containing protein 7	BRD7	651	74.092	6.39	5	1	4	1	0	1	5	1	1	1	0	1	-	-	-	-	-	-
P60709	Actin, cytoplasmic 1	β-actin/ACTB	375	41.71	5.48	35	8	20	8	63	8	44	12	31	12	87	12	54	17	47	17	95	17
Q9H8M2	Bromodomain-containing protein 9	BRD9	597	66.958	6.19	8	3	3	3	7	3	8	3	3	3	7	3	11	4	4	4	7	4

Table S2. Identification details of the SWI/SNF remodeling complex subunits identified by nano-LC–MS/MS by searching the Homo sapiens UniProtKB/Swiss-Prot database in triplicate injections of A) Benzonase-treated and B) untreated samples. PSM, peptide-to-spectra matches.

A

						Benzonase (inj #1)						Benzonase (inj #2)						Benzonase (inj #3)					
Accession	Description	Gene names	# AAs	MW [kDa]	calc. pI	Coverage	# Peptides	# PSMs	# Unique Peptides	Score Sequest HT	# Peptides Sequest HT	Coverage	# Peptides	# PSMs	# Unique Peptides	Score Sequest HT	# Peptides Sequest HT	Coverage	# Peptides	# PSMs	# Unique Peptides	Score Sequest HT	# Peptides Sequest HT
O14497	AT-rich interactive domain-containing protein 1A	BAF250A/ARID1A	2285	241.892	6.70	20	31	56	31	166	31	19	28	50	28	156	28	26	41	70	40	174	41
P51532	Transcription activator BRG1	BRG1/SMARCA4	1647	184.53	7.88	18	29	45	29	127	29	16	23	38	23	114	23	23	37	58	37	136	37
Q92922	SWI/SNF complex subunit SMARCC1	BAF155/SMARCC1	1105	122.79	5.76	24	21	38	15	108	21	22	18	33	13	97	18	26	23	45	16	106	23
Q8TAQ2	SWI/SNF complex subunit SMARCC2	BAF170/SMARCC2	1214	132.797	5.69	14	13	21	7	54	13	12	10	18	5	48	10	22	22	35	15	59	22
Q969G3	SWI/SNF-related matrix-associated actin-dependent regulator of chromatin subfamily E member 1	BAF57/SMARCE1	411	46.621	4.88	31	10	17	10	47	10	25	9	14	9	43	9	33	11	20	11	47	11
Q96GM5	SWI/SNF-related matrix-associated actin-dependent regulator of chromatin subfamily D member 1	BAF60A/SMARCD1	515	58.196	9.25	23	8	11	8	34	8	21	6	9	6	30	6	29	12	17	10	37	12
Q92785	Zinc finger protein ubi-d4	BAF45D/DPF2	391	44.127	6.33	37	8	11	8	34	8	37	8	11	8	34	8	49	13	22	13	34	13
Q12824	SWI/SNF-related matrix-associated actin-dependent regulator of chromatin subfamily B member 1	BAF47/SMARCB1	385	44.113	6.23	22	7	10	7	24	7	19	6	7	6	18	6	25	7	11	7	23	7
O96019	Actin-like protein 6A	BAF53A/ACTLA	429	47.43	5.60	17	4	7	4	20	4	17	4	6	4	20	4	17	4	7	4	20	4
Q15532	Protein SSXT	SS18	418	45.899	6.46	3	2	3	2	7	2	3	1	2	1	5	1	7	3	6	3	11	3
Q8NFD5	AT-rich interactive domain-containing protein 1B	BAF250B/ARID1B	2236	235.974	6.73	1	1	1	1	2	1	-	-	-	-	-	-	2	3	3	2	2	3
Q9NPI1	Bromodomain-containing protein 7	BRD7	651	74.092	6.39	-	-	-	-	-	-	-	-	-	-	-	-	-	-	-	-	-	-
P60709	Actin, cytoplasmic 1	β-actin/ACTB	375	41.71	5.48	52	18	75	7	212	18	50	16	63	6	191	16	54	21	95	8	225	21
Q9H8M2	Bromodomain-containing protein 9	BRD9	597	66.958	6.19	-	-	-	-	-	-	-	-	-	-	-	-	2	1	1	1	0	1

B

						Untreated (inj #1)						Untreated (inj #2)						Untreated (inj #3)					
Accession	Description	Gene names	# AAs	MW [kDa]	calc. pI	Coverage	# Peptides	# PSMs	# Unique Peptides	Score Sequest HT	# Peptides Sequest HT	Coverage	# Peptides	# PSMs	# Unique Peptides	Score Sequest HT	# Peptides Sequest HT	Coverage	# Peptides	# PSMs	# Unique Peptides	Score Sequest HT	# Peptides Sequest HT
O14497	AT-rich interactive domain-containing protein 1A	BAF250A/ARID1A	2285	241.892	6.70	19	31	52	30	155	31	18	27	47	27	145	27	21	36	59	35	161	36
P51532	Transcription activator BRG1	BRG1/SMARCA4	1647	184.53	7.88	22	31	53	31	146	31	19	26	42	26	125	26	24	39	63	24	150	39
Q92922	SWI/SNF complex subunit SMARCC1	BAF155/SMARCC1	1105	122.79	5.76	24	22	30	16	85	22	21	20	26	15	78	20	26	25	39	18	89	25
Q8TAQ2	SWI/SNF complex subunit SMARCC2	BAF170/SMARCC2	1214	132.797	5.69	18	15	19	9	48	15	16	13	16	8	43	13	23	23	32	16	50	23
Q969G3	SWI/SNF-related matrix-associated actin-dependent regulator of chromatin subfamily E member 1	BAF57/SMARCE1	411	46.621	4.88	21	10	18	10	52	10	21	10	17	10	49	10	29	13	21	13	55	13
Q96GM5	SWI/SNF-related matrix-associated actin-dependent regulator of chromatin subfamily D member 1	BAF60A/SMARCD1	515	58.196	9.25	16	9	14	9	42	9	14	7	11	7	36	7	26	14	22	11	45	14
Q92785	Zinc finger protein ubi-d4	BAF45D/DPF2	391	44.127	6.33	43	10	15	10	48	10	43	10	14	10	46	10	50	14	21	14	48	14
Q12824	SWI/SNF-related matrix-associated actin-dependent regulator of chromatin subfamily B member 1	BAF47/SMARCB1	385	44.113	6.23	34	9	11	9	32	9	34	9	11	9	32	9	34	10	14	10	34	10
O96019	Actin-like protein 6A	BAF53A/ACTLA	429	47.43	5.60	23	6	8	6	23	6	23	6	8	6	23	6	23	6	9	6	26	6
Q15532	Protein SSXT	SS18	418	45.899	6.46	7	2	2	2	6	2	7	2	2	2	6	2	7	3	4	3	9	3
Q8NFD5	AT-rich interactive domain-containing protein 1B	BAF250B/ARID1B	2236	235.974	6.73	1	2	2	1	5	2	-	-	-	-	-	-	2	3	3	2	5	3
Q9NPI1	Bromodomain-containing protein 7	BRD7	651	74.092	6.39	5	1	4	1	0	1	-	-	-	-	-	-	-	-	-	-	-	-
P60709	Actin, cytoplasmic 1	β-actin/ACTB	375	41.71	5.48	46	13	34	5	93	13	44	12	31	5	87	12	52	16	44	5	95	16
Q9H8M2	Bromodomain-containing protein 9	BRD9	597	66.958	6.19	8	3	3	3	7	3	8	3	3	3	7	3	11	4	4	4	7	4

Table S3. Identification details of proteins identified in control IgG IP sample by nano-LC–MS/MS.

Accession	Description	Gene names	# AAs	MW [kDa]	calc. pI	Coverage	# Peptides	# PSMs	# Unique Peptides	Score Sequest HT	# Peptides Sequest HT
P35527	Keratin, type I cytoskeletal 9 OS=Homo sapiens GN=KRT9 PE=1 SV=3	KRT9	623	62.027	5.24	60	25	57	25	220	25
P13645	Keratin, type I cytoskeletal 10 OS=Homo sapiens GN=KRT10 PE=1 SV=6	KRT10	584	58.792	5.21	34	16	42	15	156	16
P04264	Keratin, type II cytoskeletal 1 OS=Homo sapiens GN=KRT1 PE=1 SV=6	KRT1	644	65.999	8.12	36	19	42	17	139	19
P00760	Cationic trypsin OS=Bos taurus PE=1 SV=3	PRSS1	246	25.769	8.07	21	3	24	3	114	3
P35908	Keratin, type II cytoskeletal 2 epidermal OS=Homo sapiens GN=KRT2 PE=1 SV=2	KRT2	639	65.393	8	25	11	15	10	50	11
O14733-2	Isoform 2 of Dual specificity mitogen-activated protein kinase kinase 7 [OS=Homo sapiens]	MAP2K7	462	51.848	9.22	18	5	9	5	36	5
Q5XQN5	Keratin, type II cytoskeletal 5 OS=Bos taurus GN=KRT5 PE=1 SV=1	KRT5	601	62.898	7.81	4	2	4	1	13	2
P07477	Trypsin-1 [OS=Homo sapiens]	PRSS1	247	26.541	6.51	4	1	3	1	8	1
P02533	Keratin, type I cytoskeletal 14 OS=Homo sapiens GN=KRT14 PE=1 SV=4	KRT14	472	51.529	5.16	6	2	2	1	7	2
P08779	Keratin, type I cytoskeletal 16 OS=Homo sapiens GN=KRT16 PE=1 SV=4	KRT16	473	51.236	5.05	6	2	2	1	7	2
P02768	Serum albumin OS=Homo sapiens GN=ALB PE=1 SV=2	ALB	609	69.321	6.28	7	2	2	2	6	2
Q86YZ3	Hornerin OS=Homo sapiens GN=HRNR PE=1 SV=2	HRNR	2850	282.228	10.04	1	2	2	2	6	2
P01764	Ig heavy chain V-III region 23 [OS=Homo sapiens]	IGHV3-23	117	12.574	8.28	9	1	2	1	6	1
P08263	Glutathione S-transferase A1 [OS=Homo sapiens]	GSTA1	222	25.615	8.88	12	1	1	1	3	1
P35637-1	RNA-binding protein FUS [OS=Homo sapiens]	FUS	526	53.394	9.36	5	1	1	1	3	1
P38159-1	RNA-binding motif protein, X chromosome [OS=Homo sapiens]	RBMX	391	42.306	10.05	3	1	1	1	3	1
O14920-1	Inhibitor of nuclear factor kappa-B kinase subunit beta [OS=Homo sapiens]	IKBKB	756	86.509	5.78	5	1	2	1	0	1
Q12840	Kinesin heavy chain isoform 5A [OS=Homo sapiens]	KIF5A	1032	117.306	5.9	2	1	1	1	0	1

Table S4. List of ChIP-seq experiments of proteins used in this study.

Data	Organism	GEO Accession Number	Cell type	Reference
CTCF	Hg18	GSM325897	HeLa	Cuddapah et al., 2009
CTCF	Hg19	GSM733785	HeLa-S3	ENCODE Accession ENCFF768OPK
CTCF	Hg19	GSM749739	HeLa-S3	ENCODE AccessionENCFF830KSV
CTCF	Hg19	GSM822285	HeLa-S3	ENCODE Accession ENCFF002CSN
SMARCA4 (BRG1)	Hg18	GSM601399	HeLa-S3	Euskirchen et al., 2011
SMARCB1	Hg19	GSM935635	HeLa-S3	ENCODE Accession ENCFF002CSN
SMARCC2	Hg19	GSM935638	HeLa-S3	ENCODE Accession ENCFF002CRU
CTCF	Hg19	GSM733719	K562	ENCODE Accession ENCFF002CEL
CTCF	Hg19	GSM822311	K562	ENCODE Accession ENCFF002DBD
CTCF	Hg19	GSM935407	K562	ENCODE Accession ENCFF738TKN
CTCF	Hg19	GSM1010820	K562	ENCODE Accession ENCFF085HTY
CTCF	Hg19	GSM749733	K562	ENCODE Accession ENCFF002DDJ
SMARCA4 (BRG1)	Hg19	GSE91946	K562	ENCODE Accession ENCFF197YHU
SMARCA4 (BRG1)	Hg19	GSE91896	K562	ENCODE Accession ENCFF883TOD
SMARCB1	Hg19	GSM935634	K562	ENCODE Accession ENCFF944GJH
SMARCC2	Hg19	GSE127348	K562	ENCODE Accession ENCFF114PTZ
ARID2	Hg19	GSE105731	K562	ENCODE Accession ENCFF332ICQ
BRD9	Hg19	GSE127486	K562	ENCODE Accession ENCFF666LZV
DPF2	Hg19	GSE92006	K562	ENCODE Accession ENCFF823SYE
DPF2	Hg19	GSE127533	K562	ENCODE Accession ENCFF774QKS

Table S5. List of ChIP-seq experiments of histone modifications used in this study.

Data	Organism	GEO Accession Number	Cell type	Reference
H3K4me1 (Active enhancers)	Hg19	GSM733692	K562	ENCODE Accession ENCFF721YQO
H3K4me1 (Active enhancers)	Hg19	GSM788085	K562	ENCODE Accession ENCFF564QIG
H3K27ac (Active enhancers)	Hg19	GSM733656	K562	ENCODE Accession ENCFF931VAQ
H3K4me3 (Active promoters)	Hg19	GSE96303	K562	ENCODE Accession ENCFF752ALB
H3K4me3 (Active promoters)	Hg19	GSM733680	K562	ENCODE Accession ENCFF507QPR
H3K4me3 (Active promoters)	Hg19	GSM788087	K562	ENCODE Accession ENCFF258PHY
H3K4me3 (Active promoters)	Hg19	GSM945165	K562	ENCODE Accession ENCFF779FUO
H3K36me3 (Gene body)	Hg19	GSM733714	K562	ENCODE Accession ENCFF784HLI
H3K36me3 (Gene body)	Hg19	GSM945302	K562	ENCODE Accession ENCFF473DJX
H3K79me2 (Gene body)	Hg19	GSM733653	K562	ENCODE AccessionENCFF350GQM
H3K9me3 (Inactive enhancers)	Hg19	GSM733776	K562	ENCODE Accession ENCFF323KXF
H3K27me3 (Inactive promoters)	Hg19	GSM733658	K562	ENCODE Accession ENCFF908KJV
H3K27me3 (Inactive promoters)	Hg19	GSM788088	K562	ENCODE Accession ENCFF565WMG

Table S6. Number of consensus peaks for each histone marker (A)/proteins (B) in K562 and C) HeLa-S3 cell line.

A

Data	Cell type	Number of Consensus Peaks
H3K4me1	K562	93910
H3K27ac	K562	84264
H3K4me3	K562	28962
H3K36me3	K562	65249
H3K79me2	K562	106473
H3K9me3	K562	10847
H3K27me3	K562	154015

B

Data	Cell type	Number of Consensus Peaks
CTCF	K562	35154
SMARCA4	K562	17624
SMARCB1	K562	3079
SMARCC2	K562	19106
ARID2	K562	11835
BRD9	K562	18855
DPF2	K562	19103

C

Data	Cell type	Number of Consensus Peaks
CTCF	HeLa-S3	17292
SMARCA4	HeLa-S3	12722
SMARCB1	HeLa-S3	7780
SMARCC2	HeLa-S3	2632

Table S7. Number of peaks for each factor overlapping CTCF in the K562 and Hela-S3 cell lines. N.a. not available.

Data	Peaks Overlapping CTCF (K562)	Peaks Overlapping CTCF (HeLa-S3)
SMARCA4	1719	1041
SMARCB1	282	865
SMARCC2	1568	180
ARID2	7153	n.a.
BRD9	3626	n.a.
DPF2	1260	n.a.

Table S8. A) Number of regions for active enhancers, active promoters, gene body, inactive enhancers and inactive promoters for the K562 cell line. B) Number of factors peaks overlapping CTCF and the histone markers.

A

Data	Cell type	Number of regions
Active enhancers	K562	49821
Active Promoters	K562	28821
Gene body	K562	14451
Inactive enhancers	K562	10847
Inactive promoters	K562	154015

B

Data	Cell type	Number of Peaks Overlapping Active enhancers	Number of Peaks Overlapping Active Promoters	Number of Peaks Overlapping Gene body	Number of Peaks Overlapping Inactive enhancers	Number of Peaks Overlapping Inactive enhancers
SMARCA4 overlapping CTCF	K562	590	874	8	5	12
ARID2 overlapping CTCF	K562	734	1049	35	16	76
BRD9 overlapping CTCF	K562	1153	1589	36	11	4
DPF2 overlapping CTCF	K562	509	522	4	5	2
